# Development of the Adverse Outcome Pathway (AOP): Chronic binding of antagonist to *N*-methyl-d-aspartate receptors (NMDARs) during brain development induces impairment of learning and memory abilities of children

**DOI:** 10.1016/j.taap.2018.02.024

**Published:** 2018-09-01

**Authors:** Magdalini Sachana, Alexandra Rolaki, Anna Bal-Price

**Affiliations:** European Commission, Joint Research Centre, Ispra, Italy

**Keywords:** Adverse Outcome Pathway, Regulatory Toxicology, Developmental Neurotoxicity

## Abstract

The Adverse Outcome Pathways (AOPs) are designed to provide mechanistic understanding of complex biological systems and pathways of toxicity that result in adverse outcomes (AOs) relevant to regulatory endpoints. AOP concept captures in a structured way the causal relationships resulting from initial chemical interaction with biological target(s) (molecular initiating event) to an AO manifested in individual organisms and/or populations through a sequential series of key events (KEs), which are cellular, anatomical and/or functional changes in biological processes. An AOP provides the mechanistic detail required to support chemical safety assessment, the development of alternative methods and the implementation of an integrated testing strategy.

An example of the AOP relevant to developmental neurotoxicity (DNT) is described here following the requirements of information defined by the OECD Users' Handbook Supplement to the Guidance Document for developing and assessing AOPs. In this AOP, the binding of an antagonist to glutamate receptor *N*-methyl-d-aspartate (NMDAR) receptor is defined as MIE. This MIE triggers a cascade of cellular KEs including reduction of intracellular calcium levels, reduction of brain derived neurotrophic factor release, neuronal cell death, decreased glutamate presynaptic release and aberrant dendritic morphology. At organ level, the above mentioned KEs lead to decreased synaptogenesis and decreased neuronal network formation and function causing learning and memory deficit at organism level, which is defined as the AO. There are in vitro, in vivo and epidemiological data that support the described KEs and their causative relationships rendering this AOP relevant to DNT evaluation in the context of regulatory purposes.

## Highlights

• Inhibition of NMDA receptors function could result in learning and memory impairment
• Environmental chemicals are linked to learning and memory damage of children
• AOP concept guide development of DNT in vitro testing strategy
• Alternative approaches could support DNT regulatory testing

## 1 Introduction

To support a paradigm shift in regulatory toxicology testing and risk assessment by moving away from apical animal testing towards mechanistic knowledge based evaluation, the Adverse Outcome Pathway (AOP) concept has been proposed. AOP has been developed as a framework to facilitate a knowledge-based safety assessment that relies on understanding mechanisms of toxicity, rather than simply observing its AO. This framework helps to organize the existing information and data across multiple levels of biological organisation and identify correlative and causal linkages between the molecular initiating event (MIE) and the key events (KEs) at molecular, cellular, tissue, organism or population level, that when sufficiently perturbed by chemical exposures, result in AOs (Ankley et al., 2010; OECD, 2017). Therefore, the AOP framework provides means to adapt mechanistic understanding for regulatory decision making by consolidating, managing and exchanging knowledge between the research and regulatory communities (Delrue et al., 2016; Vinken et al., 2017).

**Fig. 1:**
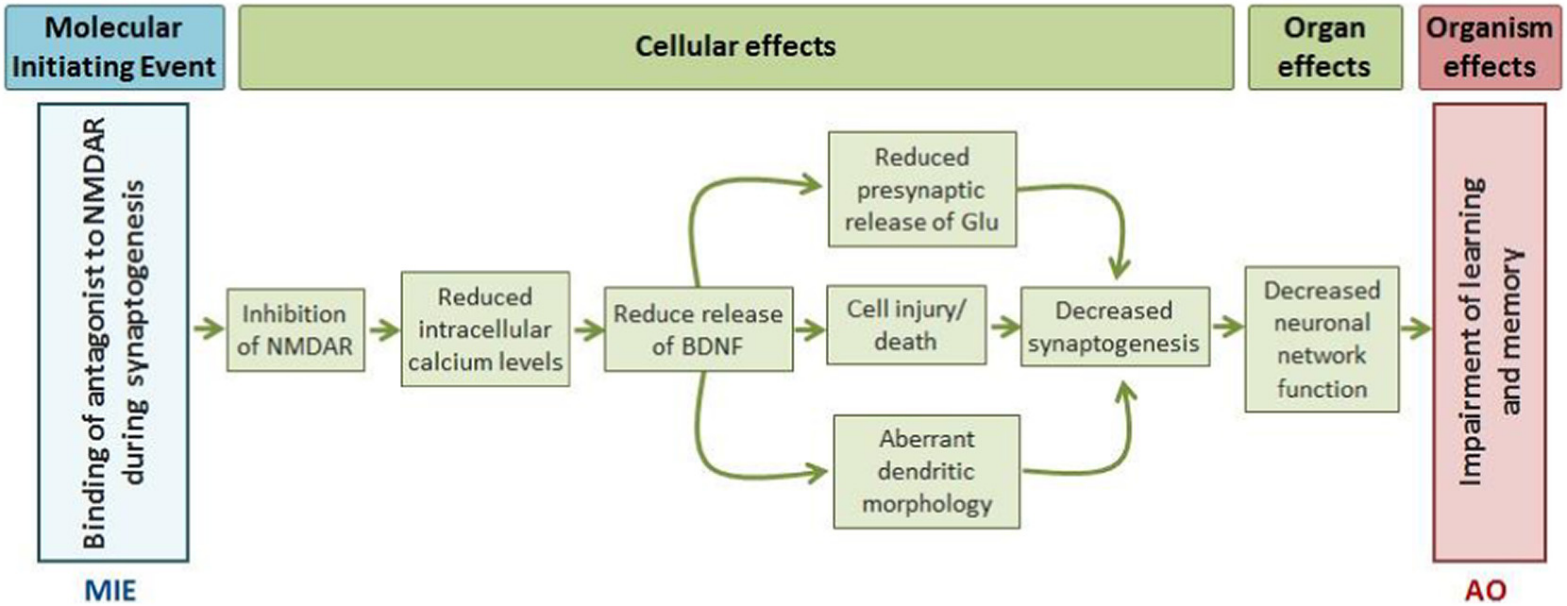
Graphical representation of the AOP. Key events at the cellular, organ and organism level triggered by binding of an antagonist to the NMDA receptor (Molecular Initiating Event) and resulting in the adverse outcome (AO), defined as impairment of learning and memory.

A variety of molecular and cellular processes is known to be crucial to proper development and function of the central nervous systems (CNS). However, there are relatively few examples of well-documented pathways with comprehensive understanding of causally linked MIEs and KEs that result in AOs in the developing brain. The functional and structural complexity of the CNS, coupled with the dynamics of brain development, suggests that a broad array of MIEs may trigger the same adverse neurological outcome and on contrary the various AOs can be caused by the same MIEs. This complexity of brain development, including different susceptibility to toxicity induced by the same chemical at different developmental windows creates a real challenge for AOP development relevant to DNT evaluation (Bal-Price et al., 2015b). Currently, in the DNT field, there are only a few DNT AOPs developed. Here, we describe the AOP developed for impairment of learning and memory processes in children (AO) caused by inhibition of glutamate receptor *N*-methyl-d-aspartate (NMDAR) function when it takes place during synaptogenesis. It is well documented in the existing literature that cognitive processes rely on physiological functioning of the NMDAR (reviewed in Rezvani, 2006; Hansen et al., 2017). In this AOP, binding of an antagonist to NMDAR was defined as MIE that causes inhibition of NMDAR function leading to reduced intracellular level of calcium, followed by reduced levels of brain derived neurotrophic factor (BDNF), reduced presynaptic glutamate release, aberrant dendritic morphology, increased cell death, decreased synaptogenesis and neuronal network formation and function resulting in impairment of learning and memory in children that was defined as adverse outcome (AO) (Fig. 1).

Damage or destruction of neurons by chemical compounds during development when they are in the process of synapses formation, integration and formation of neuronal networks will derange the organisation and function of these networks, thereby setting the stage for subsequent impairment of learning and memory. Indeed, learning-related processes require neuronal networks to detect correlations between events in the environment and store these as changes in synaptic strength (Abbott and Nelson, 2000). Long-term potentiation (LTP) and long-term depression (LTD) are two fundamental processes involved in cognitive functions (Abbott and Nelson, 2000; Malenka and Bear, 2004), which respectively, strengthen synaptic inputs that are effective at depolarizing the postsynaptic neuron and weaken synaptic inputs, thus reinforcing activation of useful pathways in the brain. A series of important findings suggest that the biochemical changes that happen after induction of LTP also occur during memory acquisition, showing temporality between the two KEs (reviewed in Lynch, 2004). Empirical support for the Key Event Relationships (KERs) of this AOP is based mainly on data published after exposure to lead (Pb^2+^) referring to in vitro, in vivo and epidemiological studies. It is well known and documented that Pb^2+^ is a potential inhibitor of the NMDA receptor that plays an important role in brain development and cognition. Chronic Pb^2+^ exposure inhibits NMDA receptor function, followed by a described cascade of KEs at the cellular and tissue level, causing impairments of nerve communication in the brain responsible for deficit in synaptic plasticity, and finally resulting in learning and memory impartment (Toscano and Guilarte, 2005). There is evidence supporting a link between exposure to Pb^2+^ and learning and memory impairment coming not only from experimental data (in vivo or in vitro) but also from cohorts, epidemiological studies discussed in the last KER of the present AOP.

This AOP is the first one relevant to DNT evaluation, developed according to the guidance document on developing and assessing AOPs (OECD, 2017) including its supplement, the Users' Hand book (OECD, 2016a) and submitted to the AOP-Wiki (https://aopwiki.org/aops/13), an AOP repository within the OECD programme on AOP Development and Assessment. The present concise version presents data in tabular format and includes recently published bibliography. Furthermore, the sections dealing with the weight of evidence (WoE) evaluation of KEs, KERs and overall AOP have been extensively revised as the way to assemble and assess the degree of confidence that supports AOPs has evolved and significantly as more experience has been gained in this field.

## 2 Structure and major principles of AOP development

As mentioned above the AOP concept describes a sequence of measurable KEs that are correlatively or causally linked (Fig. 1), originating from a MIE in which a chemical interacts with a biological target (OECD, 2016a), triggering the first KE downstream. The initiated by the MIE sequential series of KEs includes molecular, cellular, anatomical and/or functional changes in biological systems and ultimately results in a KE at tissue level leading to an AO of regulatory relevance for human health or eco-toxicological risk assessment.

Understanding physiological pathways is the basis for describing the perturbations that occur following chemical exposure. The observed changes of biological state that should be measurable at different levels of biological organisation (cellular, tissue, organ or organism) as well as its general role in biology should be covered in the description of KEs (including specialised KEs such as MIE and AO). KERs should assemble and organise evidence that would facilitate establishment of the scientific basis permitting extrapolation of the state of the KE downstream from the measured KE upstream.

By definition AOPs are not chemical specific and the described KEs should be independent from any specific chemical initiator (Villeneuve et al., 2014). However, an empirical support for KER description refers to the experimental data derived from exposure to chemicals to be able to illustrate understanding of the patters of biological responses between identified KEs based on reviewed literature. It is important that there are studies available where the compounds have been clinically proven to trigger the identified AO, supporting the developed AOP. For example, in the present AOP the empirical support for KERs is mainly based on experimental data obtained after exposure to Pb^2+^ as reference chemical since there are abundant in vivo, in vitro and epidemiological studies suggesting that cognitive deficit in children is linked to chronic exposure to this heavy metal. However, any chemical that will bind and block NMDA receptor function, triggering the described cascade of KEs during synaptogenesis will be relevant to this AOP according to the rule that AOPs should be chemically agnostic.

**Fig. 2:**
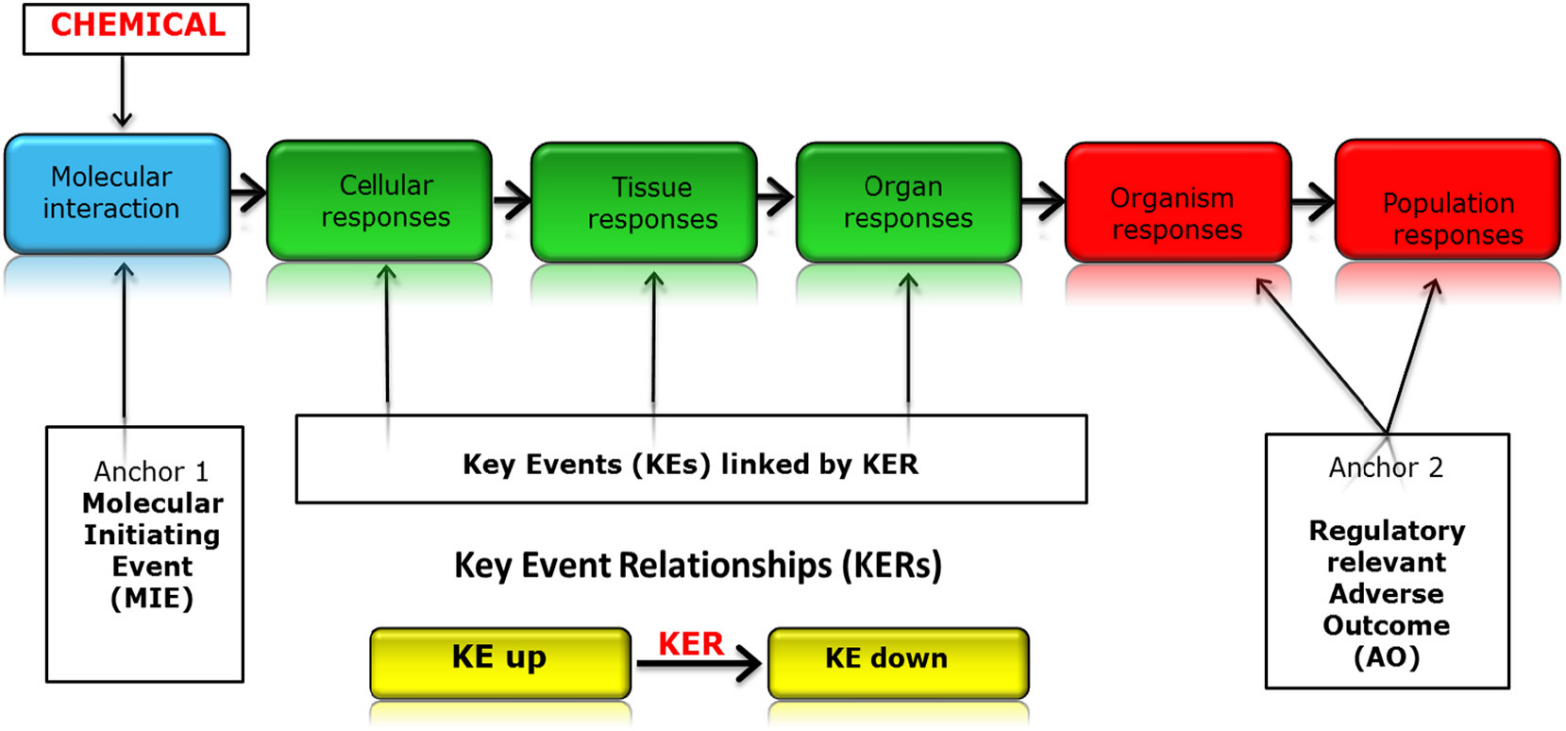
A schematic representation of the Adverse Outcome Pathway (AOP) framework. An AOP is triggered by a Molecular Initiating Event (MIE), an initial interaction with a biological target (Anchor 1) that leads to a sequential cascade of cellular, tissue and organ responses (Key Events), linked to each other by key event relationship (KER) to result in an adverse outcome (AO) of regulatory relevance.

In principle, AOPs are defined as linear, non-branching sequences of KEs, linking MIE to AO (Fig. 2) despite of the complex feed-back loops, defense mechanisms, modifying factor that contribute to the interactions between KEs. These can be described in the text of KERs.

However, for this AOP was justified to present three KEs (neuronal cell death, decreased presynaptic release of glutamate and aberrant dendritic morphology), causally linked to the MIE and AO, happening simultaneously since they are acting together leading the downstream KE (decreased synaptogenesis). This representation is more practical both for the development and use of the AOP than breaking those multiple highly related pathways into separate AOPs.

The whole AOP is evaluated by weight of evidence (WoE), applying modified Bradford-Hill considerations referring to the biological plausibility of KERs, essentiality of KEs and empirical support for KERs (Becker et al., 2015; Bal-Price et al., 2017). Biological plausibility of KERs describes how well the mechanistic relationship between KE upstream and KE downstream is understood with respect to current knowledge. Essentiality should refer to experimental support proving evidence that blockage of any KE upstream will prevent KEs downstream or the AO. Empirical support for KER should assess concordance of the doses and time at which the upstream and downstream KEs are observed, and incidence concordance. The overall assessment of the level of confidence in the developed AOP is based on the essentiality experimental data for each KE, the biological plausibility and the empirical support for KERs (see Section 4).

## 3 Description of the identified key event relationships (KERs)

### 3.1 KER: binding of antagonist to NMDA receptors (MIE) leads to Inhibition of NMDARs function

#### 3.1.1 KE upstream: binding of antagonist to NMDARs

Due to its physiological and pharmacological properties, glutamate (Glu) activates three classes of ionotropic receptors named: α-amino-3-hydroxy-5-methyl-4-isoazolepropionic acid (AMPARs), 2-carboxy-3-carboxymethyl-4-isopropenylpyrrolidine (kainate receptors, KARs) and *N*-methyl-d-aspartate (NMDA receptors, NMDARs). NMDA receptor, is composed of two NR1 subunits that are combined with either two NR2 (NR2A, NR2B, NR2C NR2D) subunits and less commonly are assembled together with a combination of NR2 and NR3 (NR3A, NR3B) subunits (reviewed in Traynelis et al., 2010; Hansen et al., 2017). To be activated NMDA receptors require simultaneous binding of both Glu to NR2 subunits and of glycine to either NR1 or NR3 subunits that provide the specific binding sites named extracellular ligand-binding domains (LBDs). NMDAR can also be activated indirectly through initial activation of KA/AMPARs. Binding of agonist to KA/AMPARs results in ion influx (mainly of Na^+^ and to a small extend of K^+^) and glutamate release from excitatory synaptic vesicles causing depolarization of the postsynaptic neuron (Dingledine et al., 1999). Upon this depolarization the Mg^2+^ block is removed, allowing sodium, potassium, and importantly calcium ions to enter into a cell. At positive potentials, NMDARs then show maximal permeability (i.e., large outward currents can be observed under these circumstances). Due to the time needed for the Mg^2+^ removal, NMDARs activate more slowly, having a peak conductance long after the KA/AMPAR peak conductance takes place. It is important to note that NMDARs conduct currents only when Mg^2+^ block is relieved, glutamate and glycine is bound, and the postsynaptic neuron is depolarized.

In hippocampus (critical brain region form cognitive functions), NR2A and NR2B are the most abundant NR2 family subunits. NR2A-containing NMDARs are mostly expressed synaptically, while NR2B-containing NMDARs are found both synaptically and extrasynaptically (Tovar and Westbrook, 1999).

#### 3.1.2 KE downstream: inhibition of NMDARs function

NMDA receptors, when compared to the other Glu receptors, are characterised by higher affinity for Glu, slower activation and desensitisation kinetics and higher permeability for calcium (Ca^2+^) and susceptibility to potential-dependent blockage by magnesium ions (Mg^2+^). They are involved in fast excitatory synaptic transmission and neuronal plasticity in the CNS. Ca^2+^ flux through the NMDA receptor is considered to play a critical role in pre- and post-synaptic plasticity, a cellular mechanism important for learning and memory (Barria and Malinow, 2002). The NMDA receptors have been shown also to play an essential role in the strengthening of synapses and neuronal differentiation, through long-term potentiation (LTP), and the weakening of synapses, through LTD. All these processes are important units implicated in the memory and learning processes (Barria and Malinow, 2002).

#### 3.1.3 Weight of evidence (WoE) evaluation

##### 3.1.3.1 Biological plausibility for this KER

**Fig. 3:**
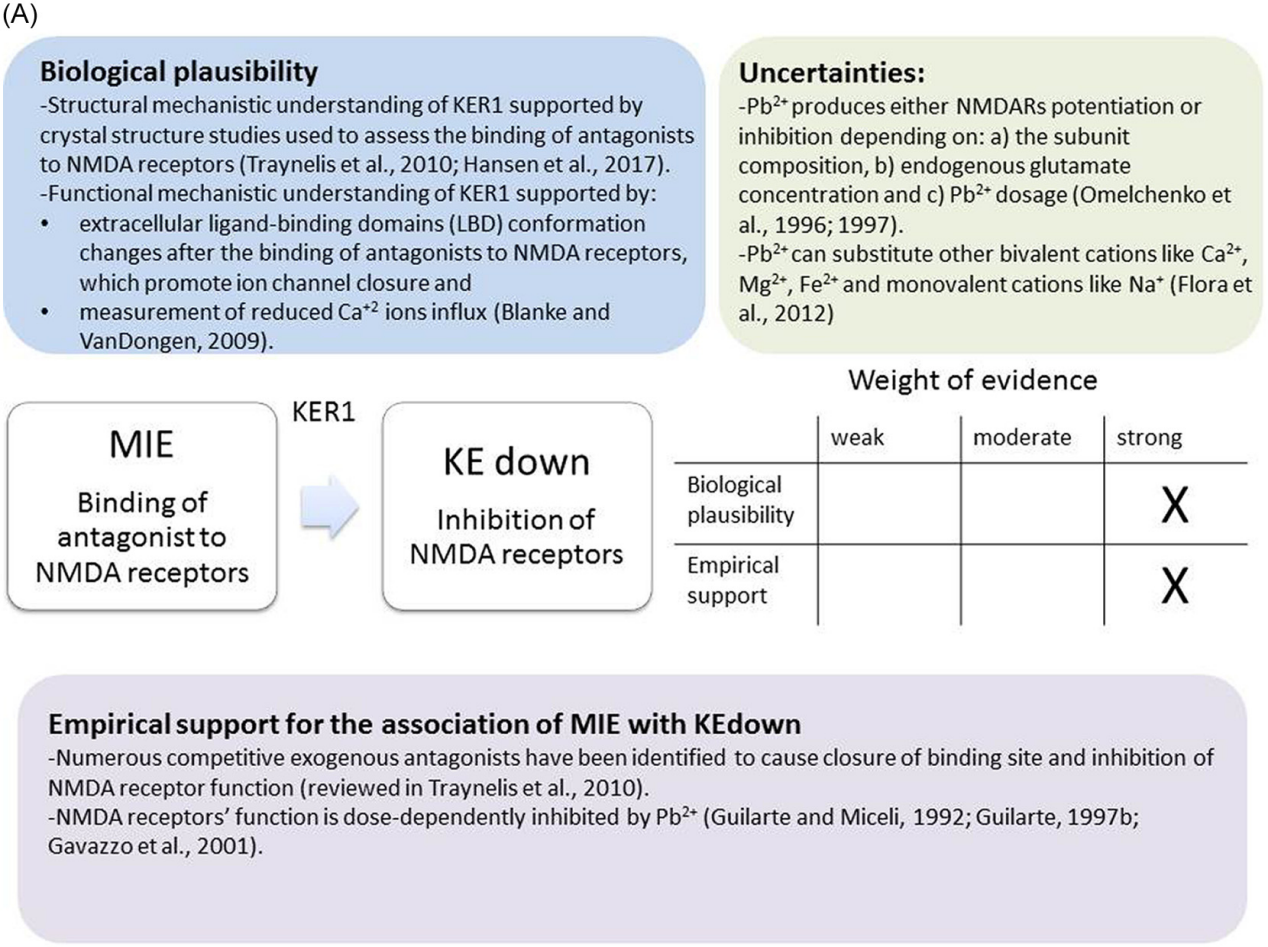

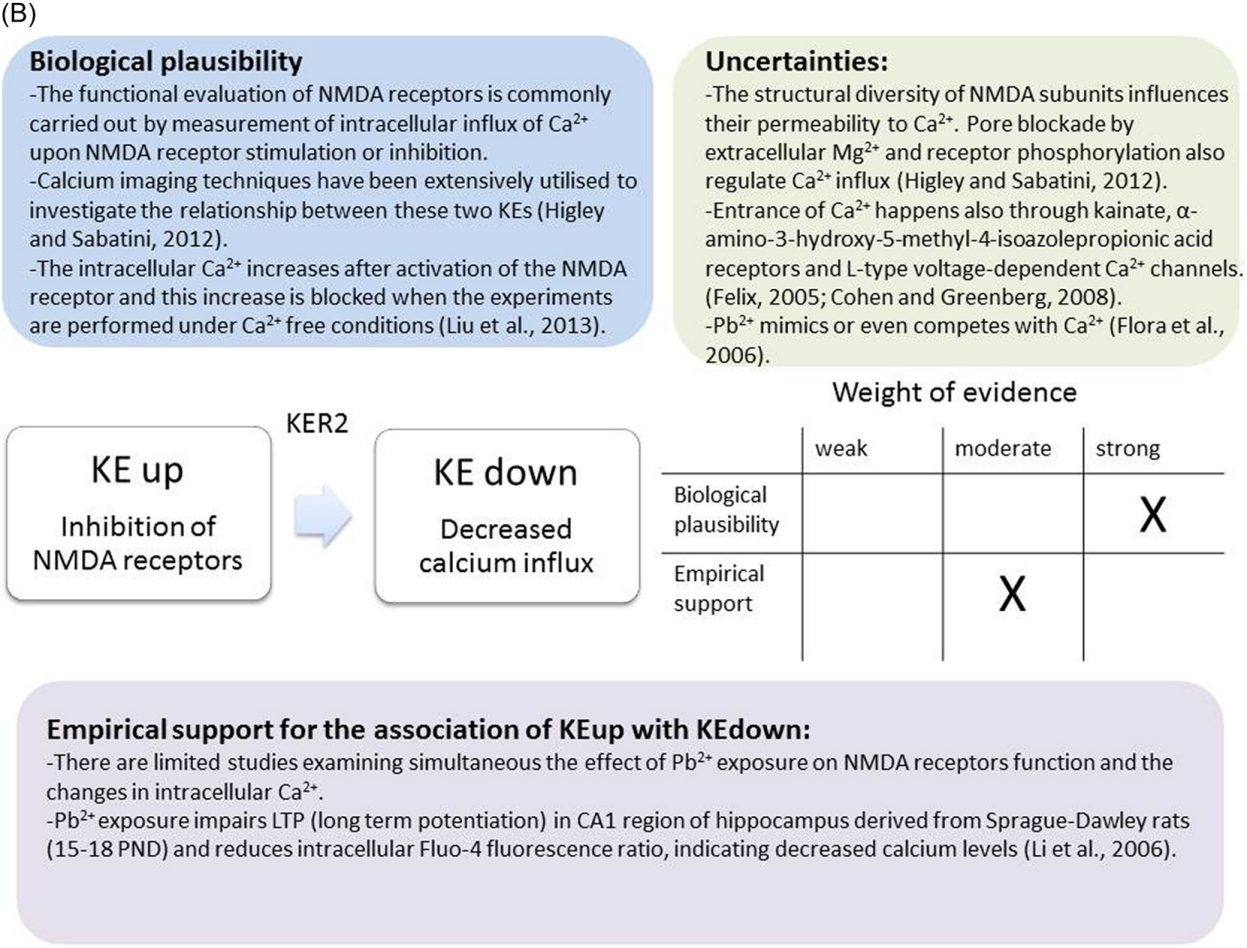

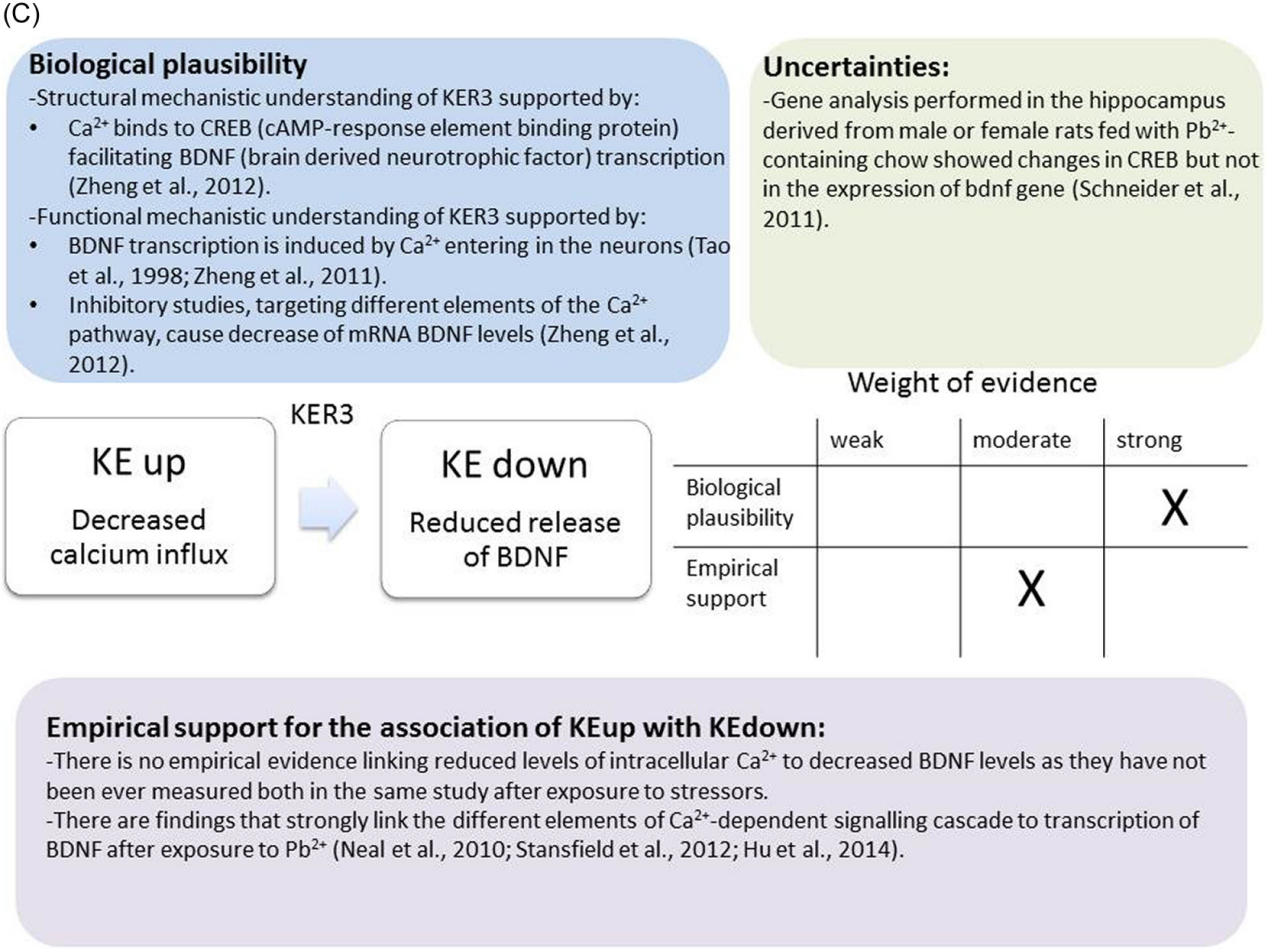

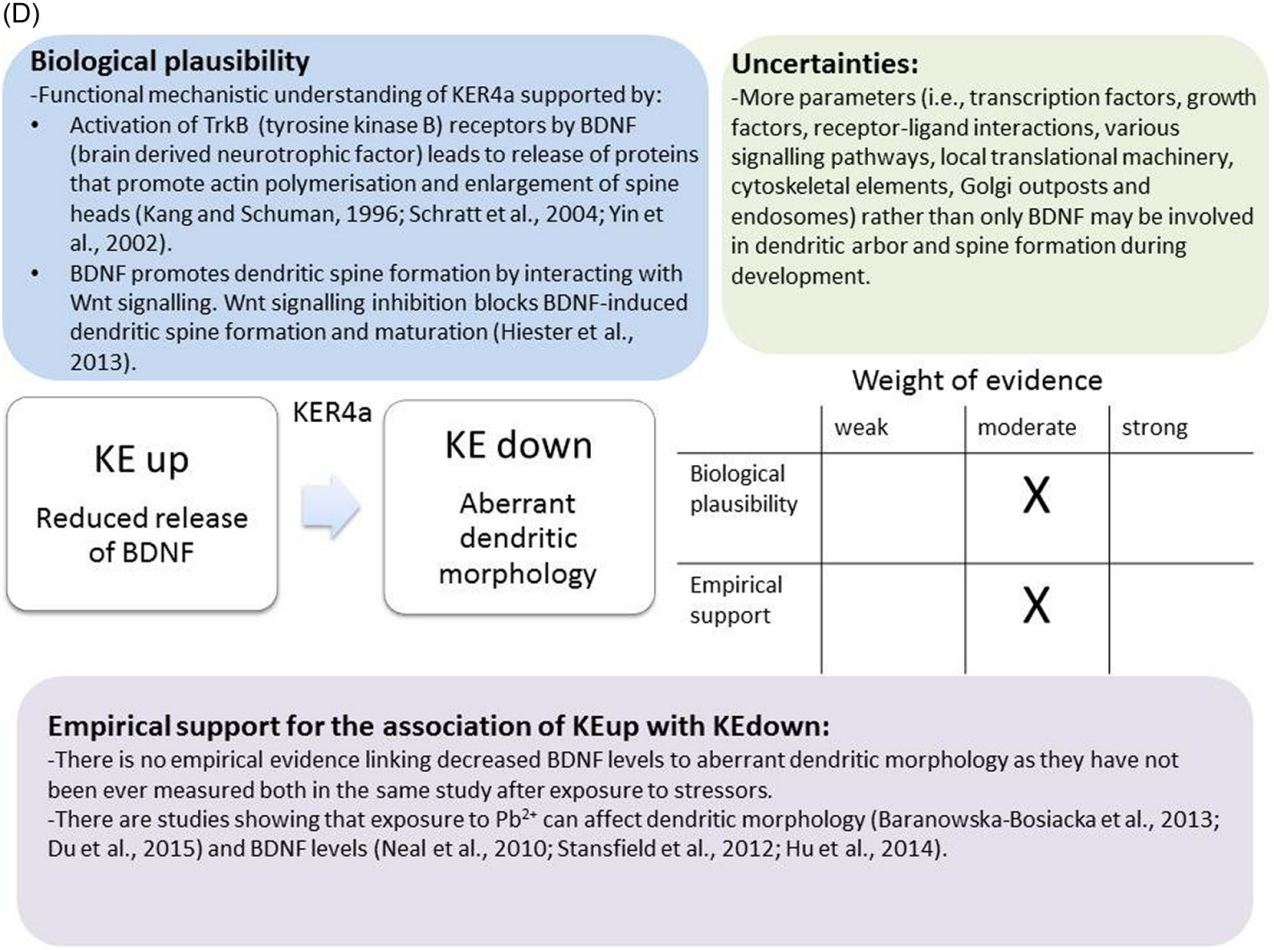

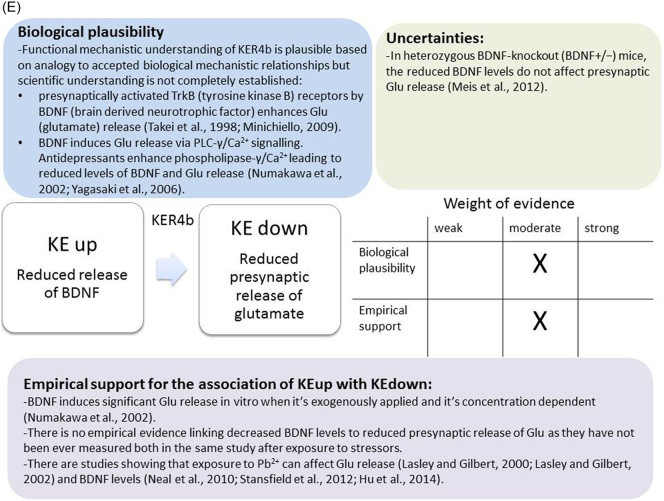

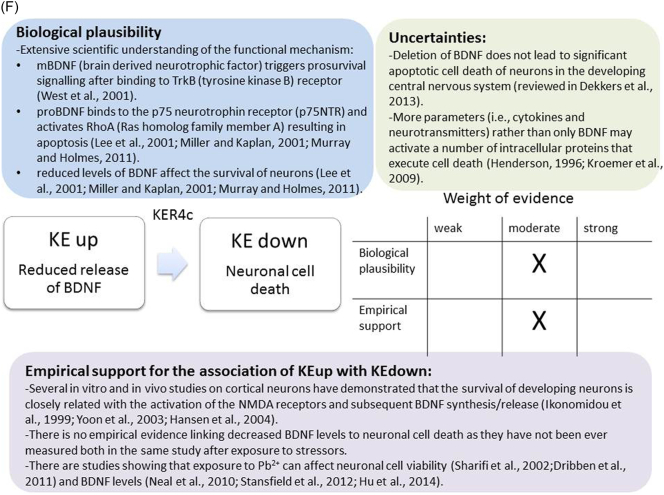

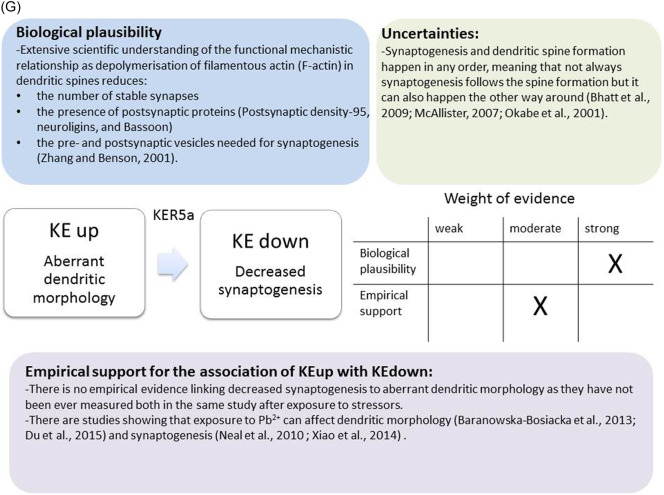

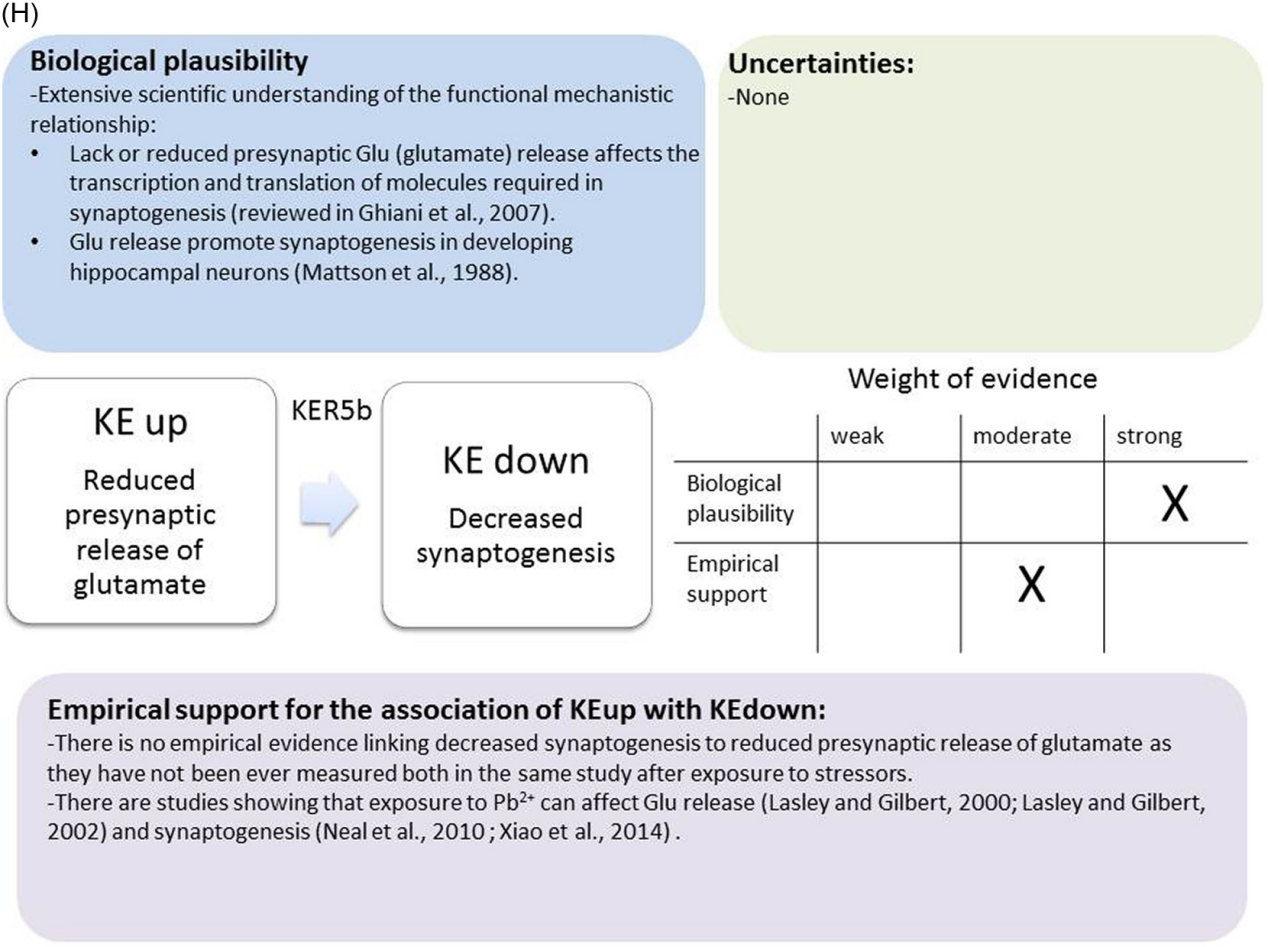

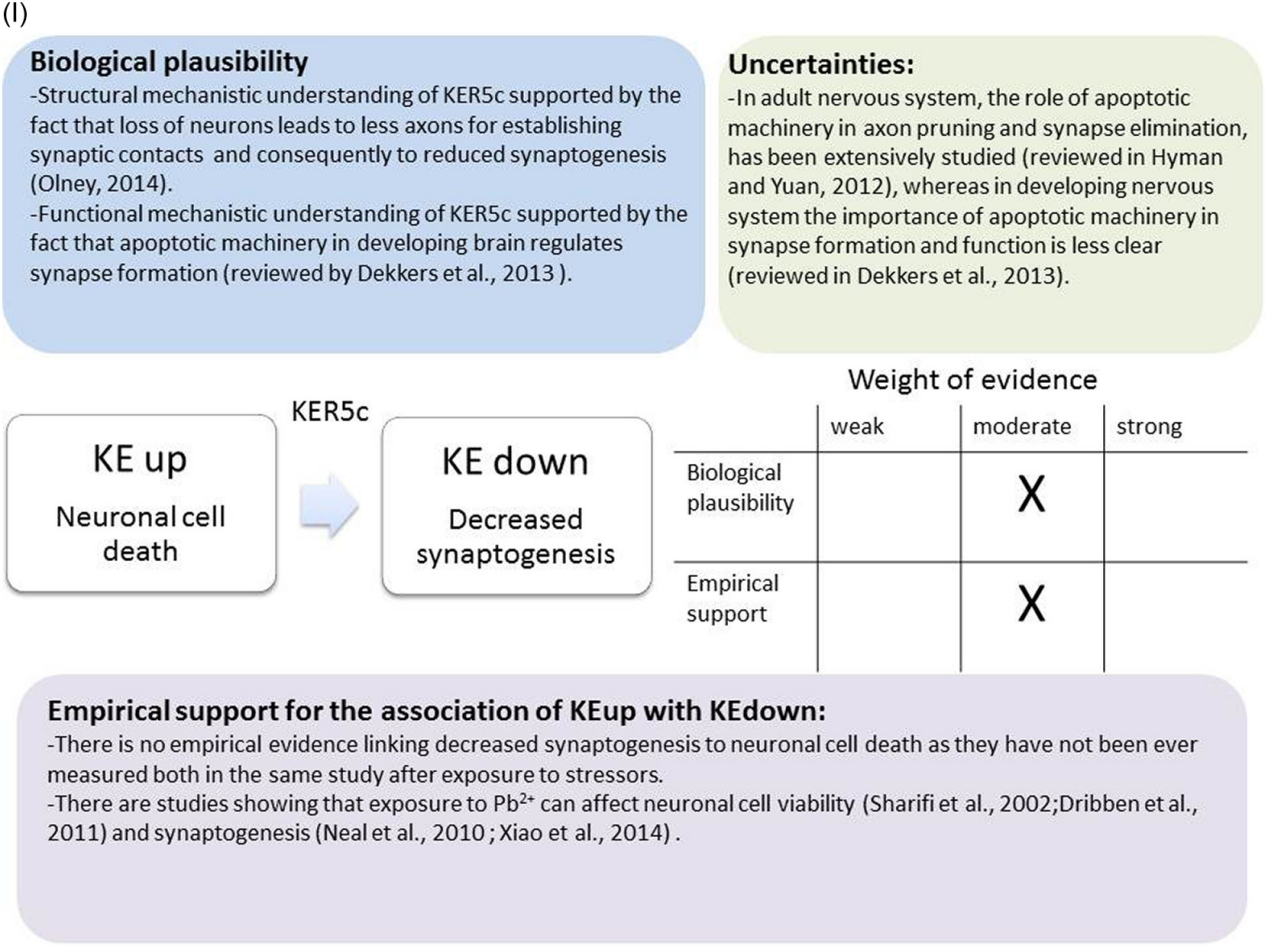

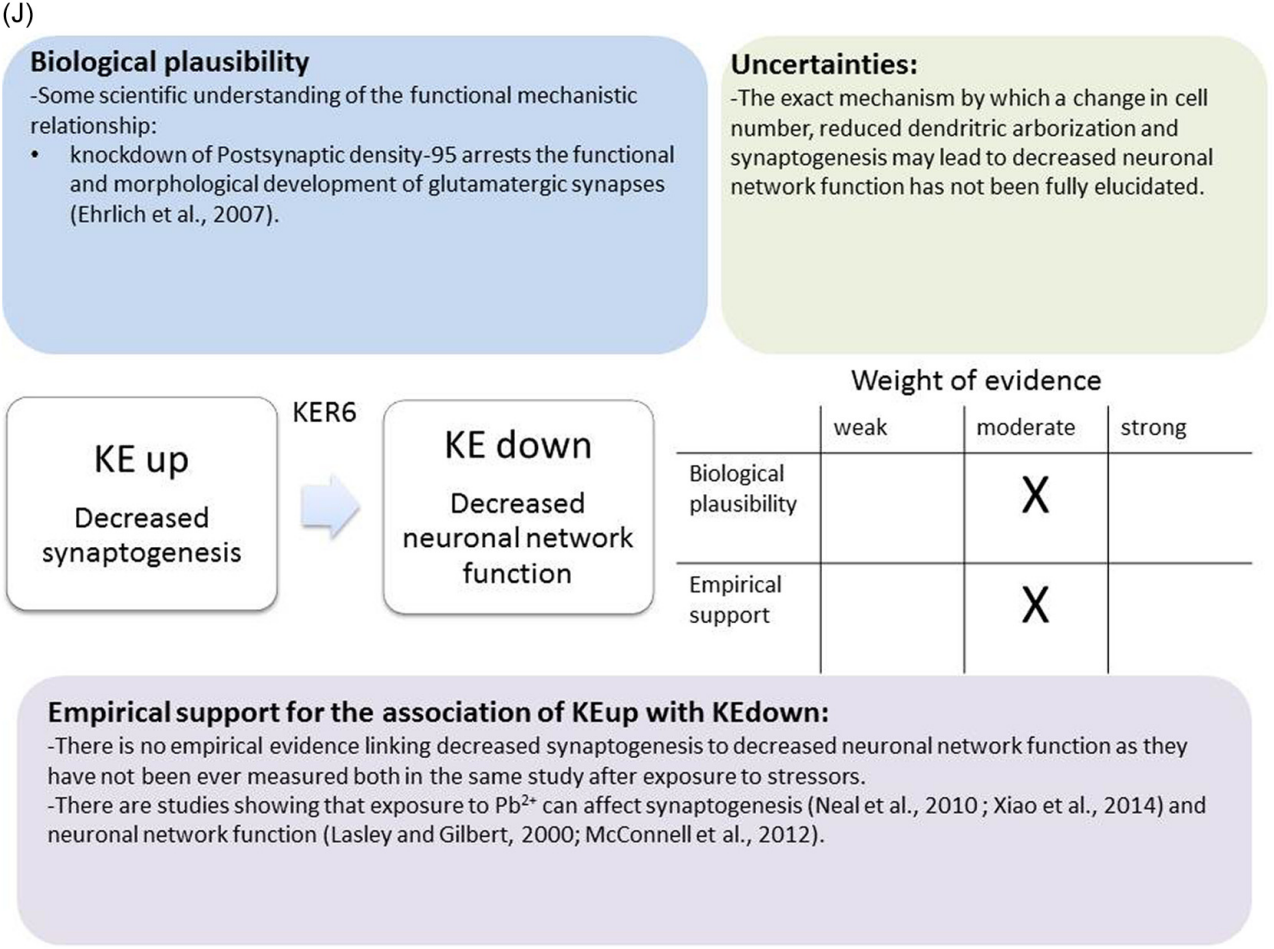

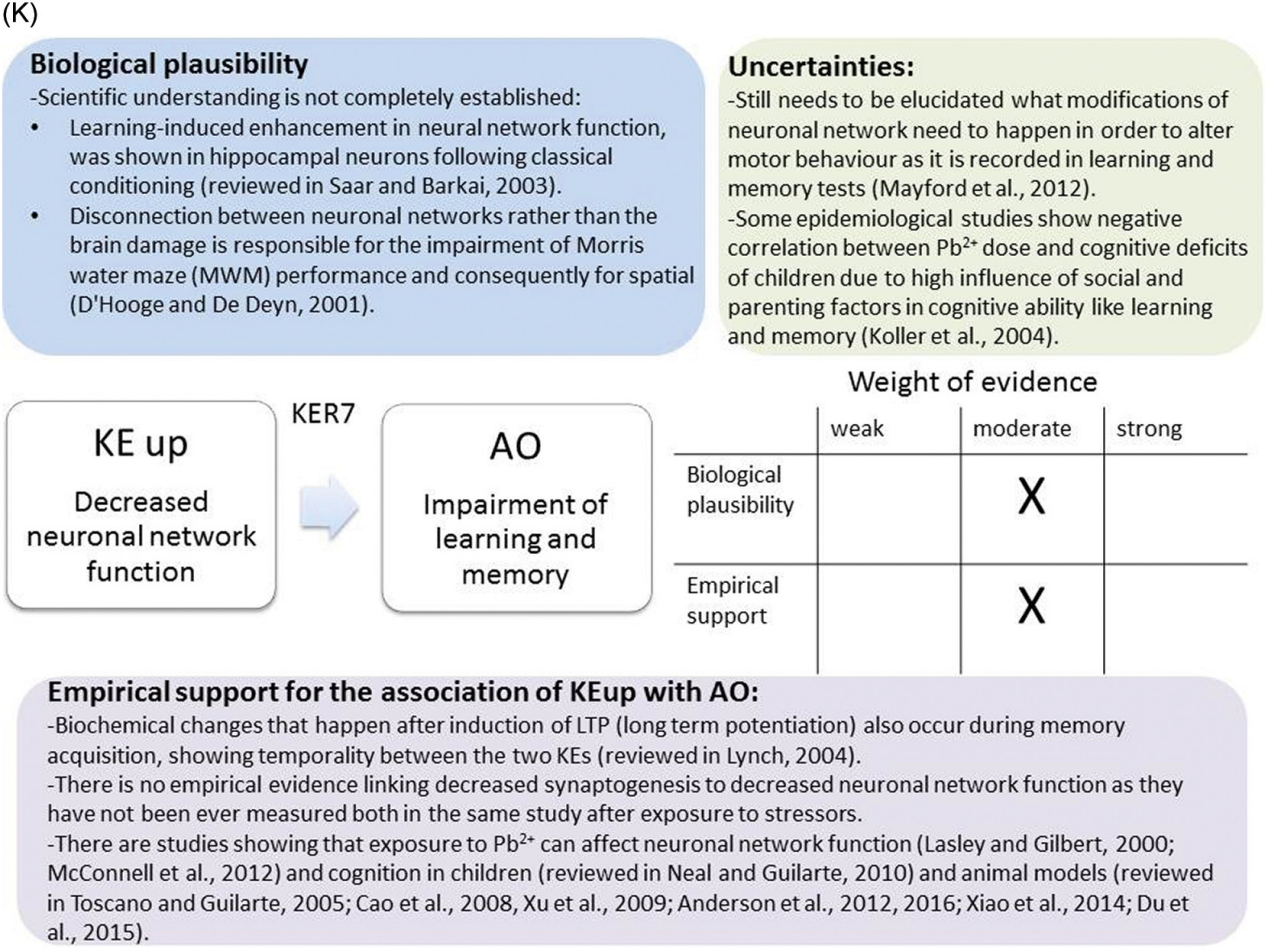
A qualitative assessments of Key Event Relationships (KERs) in the AOP triggered by binding of an antagonist to *N*-methyl-d-aspartate receptors (NMDARs) (molecular initiating event: MIE) resulting in an impairment of learning and memory (adverse outcome: AO) referring to biological plausibility, empirical support and uncertainties. (A) KER 1, antagonist binding to NMDARs results in the inhibition of NMDA receptor function; (B) KER 2, the inhibition of NMDA leads to decreased calcium influx; (C) KER 3, decreased calcium influx causes reduced release of BDNF; (D) KER 4a, reduced release of BDNF results in aberrant dendritic morphology; (E) KER 4b, reduced release of BDNF leads to reduced presynaptic release of glutamate; (F) KER 4c, reduced release of BDNF causes neuronal cell death; (G). KER 5a, aberrant dendritic morphology results in decreased synaptogenesis; (H) KER 5b, reduced presynaptic release of glutamate leads to decreased synaptogenesis; (I) KER 5c, neuronal cell death leads to decreased synaptogenesis; (J) KER 6, decreased synaptogenesis results in decreased neuronal network function; (K) KER 7, decreased neuronal network function leads to impairment of learning and memory (AO).

The weight of evidence for this KER is rated as strong (Fig. 3A). There is structural and mechanistic understanding supporting the relationship between the MIE (binding of antagonists to NMDARs) and the KE down (inhibition of NMDARs function). Crystal structure studies were applied to study the binding of antagonists/agonists to NMDA receptors (Traynelis et al., 2010; Hansen et al., 2017). Binding of antagonists to NMDARs, causes LBD conformation changes, which promote channel closure leading to reduced (or blockage) of Ca^+2^ influx. The decreased (or lack) of Ca^+2^ ions influx can be measured and is considered as a readout of decreased (or blocked) NMDAR function (Blanke and VanDongen, 2009).

##### 3.1.3.2 Empirical support for linkage

It is well understood and documented that Pb^2+^ has potent concentration-dependent inhibitory effects on the NMDA receptors function (Guilarte and Miceli, 1992; Guilarte, 1997a, Guilarte, 1997b; Gavazzo et al., 2001). These inhibitory effects of Pb^2+^ on NMDA receptors activation appear to be age and brain region specific (Guilarte, 1997b; Guilarte and Miceli, 1992). For Pb^2+^ the half maximal inhibition concentrations (IC_50_) is significantly lower in cortical membranes prepared from neonatal than from adult rats (Guilarte and Miceli, 1992). As regards the brain regions, hippocampus is more sensitive than the cerebral cortex since the IC_50_ for Pb^2+^is significantly lower in hippocampus (Guilarte and Miceli, 1992). During synaptogenesis the hippocampus appears to be particularly vulnerable to Pb^2+^ exposure as in this brain structure NMDA receptors undergo subunit specific changes during development. (Guilarte and McGlothan, 1998). Pb^2+^decreases the expression of hippocampal NR2A-subunit of NMDARs at synapses and increases targeting of NR2B-NMDARs to dendritic spines (without increased NR2B-NMDARs expression), resulting in decreased protein synthesis in dendrites that are important for learning and memory processes (Neal et al., 2011; Zhang et al., 2002).

To predict how potent an antagonist can be, the IC_50_ and the half maximal effective concentration (EC_50_) of glutamate/glycine-induced currents in NMDA receptors was measured from brain slices and cells or in recombinantly expressed receptors. Traynelis et al. (2010) summarised the IC_50_ values for competitive, non-competitive and uncompetitive antagonists in different subunits of NMDA receptors. Also quantitative evaluation of Zn^2+^ binding to the NR2 subunits have been determined (Gavazzo et al., 2008).

It is worth noting that in contrast to chronic exposure and persistent antagonism of the NMDA receptor for a period of days, acute inhibition of NMDAR function may trigger different downstream KEs, such as up-regulation of the NMDARs, resulting in an increased influx of calcium and neuronal cell death (Ikonomidou et al., 1999). Thus, it should be described as a different KER that could lead to development of different AOP.

### 3.2 KER: inhibition of NMDARs (KE upstream) leads to decreased calcium influx (KE downstream)

#### 3.2.1 KE downstream: reduced calcium influx

Under physiological conditions, the free intracellular Ca^2+^ concentration is lower (100 nM) than the extracellular Ca^2+^ concentration (1.2 mM). The latter under certain conditions may enter the cell and accumulate in the cytoplasm, cellular organelles (e.g., mitochondria and endoplasmic reticulum) and nucleus (Berridge et al., 2000).

Ca^2+^ acts as an important intracellular messenger and consequently regulates many different cellular functions (Berridge, 2012; Hagenston and Bading, 2011). Therefore,Ca^2+^ homeostasis is tightly regulated by intracellular and extracellular mechanisms (Barhoumi et al., 2010). In neurons, Ca^2+^ ions regulate many critical functions such as postsynaptic depolarisation and activation of Ca^2+^-sensitive proteins that trigger signalling pathways critical for cell physiology. Modification of the gene transcription by Ca^2+^ ions impacts long-term neurotransmitters release (reviewed in Neher and Sakaba, 2008), neuronal differentiation (Toth et al., 2016), synapse function (Wang and Peng, 2016) and cell viability (Higley and Sabatini, 2012). Thus, the Ca^2+^ that enters and accumulates in cytoplasm and nucleus is a central signalling molecule that regulates fundamental for learning and memory processes (Berridge, 2012; Hagenston and Bading, 2011).

#### 3.2.2 WoE evaluation

##### 3.2.2.1 Biological plausibility for KER

The biological plausibility of the relationship between the KEupstream Inhibition of NMDARs function and the KEdownstream Decreased Calcium influx is strong (Fig. 3B). The functional evaluation of NMDA receptors is commonly carried out by measurement of intracellular influx of Ca^2+^ upon NMDA receptor stimulation or inhibition. Calcium imaging techniques have been extensively utilised to investigate the relationship between these two KEs. Almost 15% of the current through NMDA receptors is mediated by Ca^2+^ under physiological conditions (Higley and Sabatini, 2012). However, the majority of the Ca^2+^ ions are rapidly eliminated by binding of calcium to proteins, reaching rapidly ~1 μM of free intracellular calcium concentration (Higley and Sabatini, 2012). In rat primary forebrain cultures, the intracellular Ca^2+^ increases after activation of the NMDA receptor and this increase is blocked when the experiments are performed under Ca^2+^ free conditions, demonstrating that the NMDA-evoked increase in intracellular Ca^2+^ derives from extracellular and not intracellular sources (Liu et al., 2013).

##### 3.2.2.2 Empirical support for linkage

There are a few studies examining the effect of Pb^2+^ exposure on the changes in intracellular Ca^2+^. Incubation of rat synaptosomes with Pb^2+^ stimulates the activity of calmodulin reaching the higher effect at 30 μM, whereas higher concentrations of Pb^2+^ causes inhibition (Sandhir and Gill, 1994). The Pb^2+^ IC_50_ values for inhibition of Ca^2+^ ATPase has been found to be 13.34 and 16.69 μM in calmodulin-rich and calmodulin-depleted synaptic plasma membranes, respectively. Exposure of rats to Pb^2+^ has also inhibitory effect on Ca^2+^ ATPase activity, causing increase in intra-synaptosomal Ca^2+^ (Sandhir and Gill, 1994).

Furthermore, there is evidence that Pb^2+^ exposure affects Ca^2+^ homeostasis causing alterations in the phosphorylation state of different kinases. For example, Pb^2+^ has been shown to interfere with MAPK signalling increasing the phosphorylation of both ERK1/2 and p38(MAPK) (Cordova et al., 2004). However, the findings regarding calcium/calmodulin kinase II (CamKII) activity after exposure to Pb^2+^ are not clear (Toscano and Guilarte, 2005). On one hand, Pb^2+^ has been found to cause reduction of CREB phosphorylation in the hippocampus of rats exposed during brain development (Toscano et al., 2002, Toscano et al., 2003). One the other, the levels of phosphorylation of CamKII have not been explored but only the mRNA expression levels have been studied in rat pups on PND 25 that received Pb^2+^ (180 and 375-ppm lead acetate in food for 30 days) and reached blood Pb^2+^ levels 5.8 to 10.3 μg/dl on PND 55 (Schneider et al., 2012). In this study, CamKIIα gene expression has been found to be very sensitive to Pb^2+^ exposure in the frontal cortex (Schneider et al., 2012).

Pb^2+^ (10 μM) exposure impairs LTP in CA1 region of hippocampus derived from Sprague-Dawley rats (15–18 PND) as it has been recorded by whole cell patch-clamp technique (Li et al., 2006). Pb^2+^ chronically or acutely applied, significantly reduces LTP in CA1 region of hippocampus from Wistar or Sprague-Dawley rats (30 and 60 PND) (Carpenter et al., 2002). In CA3, there have been was a dramatic difference in response as the age of animals increased. At 30 days, LTP was significantly reduced but at 60 days LTP was increased by about 30% (Carpenter et al., 2002). In the same brain structure and area (CA3), the effects of Pb^2+^ on LTP have been different in rats exposed to PND 30 and PND 60 after either perfusion of Pb^2+^ or from slices derived from rats after chronic developmental exposure to Pb^2+^ (Hussain et al., 2000). Inhibition of LTP has been recorded in CA3 area from animals sacrificed on PND 30, whereas potentiation has been measured in the same brain area derived from older animals (PND 60) with either exposure paradigm.

However, when somebody interprets results related to this KER there is need to take into consideration the following parameters, which could explain some inconsistencies: (a) the structural diversity of NMDA subunits at different windows of brain development can influence the functionality of the receptors and their permeability to Ca^2+^ (Higley and Sabatini, 2012); (b) the membrane potential due to pore blockade by extracellular Mg^2+^ and receptor phosphorylation (Higley and Sabatini, 2012); (c) the entrance of Ca^2+^ into neuronal cell that can also happen through KA and AMPA receptors but at much lower extend in compareson to NMDA receptors (Higley and Sabatini, 2012). However, AMPA receptors may also contribute to Ca^2+^ signalling during CNS development (Cohen and Greenberg, 2008); (d) Ca^2+^ entry occurs also through L-type voltage-dependent Ca^2+^ channels (L-VDCCs) (Felix, 2005), suggesting that there are more possible entrance sites for Ca^2+^ to get into the cytosol rather than only through NMDA receptors; (e) Pb^2+^ has the ability to mimic or even compete with Ca^2+^ in the CNS, accumulating in the same mitochondrial compartment as Ca^2+^ (Flora et al., 2006). So, it is possible that the reduced levels of Ca^2+^ after Pb^2+^ exposure may not be attributed only to NMDA receptor inhibition but also to the ability of this heavy metal to compete with Ca^2+^.

### 3.3 KER: decreased calcium influx (KE upstream) leads to reduced release of brain derived neurotrophic factor (BDNF) (KE downstream)

#### 3.3.1 KE downstream: reduced release of BDNF

BDNF initially is synthesised as precursor proteins (proBDNF), which is processed intracellularly to its mature form (mBDNF) after proteolytically cleaved in the synaptic cleft by plasmin, which is a protease activated by tissue plasminogen activator (tPA) (Cohen-Cory et al., 2010; Leal et al., 2017). proBDNF is constantly secreted while tPA release and mBDNF production depends on neuronal excitation (Head et al., 2009). Storage and activity-dependent release of BDNF has been demonstrated in both dendrites and axon terminals (Waterhouse and Xu, 2009).

The biological functions of mBDNF are mediated by binding to tyrosine kinase B (TrkB) receptors that lead to the activation of three major intracellular signalling pathways, including MAPK, PI3K and PLCγ1 (Soulé et al., 2006). TrkB-mediated signalling regulates gene transcription in the nucleus through the activation of several transcription factors involved in neurite outgrowth, synaptogenesis, synapse maturation and stabilization (Lu et al., 2005; Nelson and Alkon, 2014). On the other hand, proBDNF binds to the p75 neurotrophin receptor (p75NTR) and activates RhoA, a small GTPase that regulates actin cytoskeleton polymerization leading to inhibition of axonal elongation, growth cone collapse, and apoptosis (Yamauchi et al., 2004; Head et al., 2009).

#### 3.3.2 WoE evaluation

##### 3.3.2.1 Biological plausibility for this KER

There is extensive scientific understanding of the functional and structural mechanistic relationship between KEup: Decreased Calcium influx and the following KEdown: Reduced release of BDNF (Fig. 3C). BDNF transcription is induced by Ca^2+^ entering in the neurons through either L type voltage gated calcium channel (L-VGCC) (Tao et al., 1998) or NMDA receptor (Tabuchi et al., 2000; Zheng et al., 2011). BDNF IV that is the most studied among its different exons has been shown to bind three Ca^2+^ elements within the regulatory region (Zheng et al., 2012). Ca^2+^ binds to CREB facilitating BDNF transcription. The activation of the relevant transcription factor is triggered by the initial activation of CaM kinase, cAMP/PKA and Ras/ERK1/2 pathways mediated by the elevated intracellular Ca^2+^. Inhibitory studies, targeting different elements of these pathways, show that Ca^2+^ can also decrease mRNA BDNF levels (Zheng et al., 2012).

Increase of intracellular Ca^2+^ levels phosphorylates MeCP2, which inactivates its repressor function and permits the transcription of BDNF exon IV (Tao et al., 2009; Zhou et al., 2006). Indeed, NMDA receptor activation has been shown to upregulate BDNF transcripts containing exon IV not only via Ca^2+^-dependent CREB but also through Ca^2+^ activation of MeCP2 transcription (Jiang et al., 2005; Zheng et al., 2011).

##### 3.3.2.2 Empirical support for linkage

There is no direct evidence linking reduced levels of intracellular Ca^2+^ to decreased BDNF levels as they have not been ever measured both in the same study after exposure to stressors. However, there are findings that strongly link the Ca^2+^-dependent signalling cascade to transcription of BDNF.

Pb^2+^ decreases the ratio of phosphorylated versus total MeCP2 and consequently MeCP2 maintains its repressor function and prevents BDNF exon IV transcription (Stansfield et al., 2012). MeCP2 gene expression in the frontal cortex is very sensitive to Pb^2+^ exposure while in the hippocampus, the same gene is affected only at the higher exposure of rat pups (PND 55) with blood Pb^2+^ levels 5.8 to 10.3 μg/dl (Schneider et al., 2012). The doses of Pb^2+^ that result in learning and LTP deficits in rats cause also the decrease in phosphorylation of CREB in cerebral cortex at PND 14 and the reduction in phosphorylation state of CREB in both cortex and hippocampus at PND 50 (Toscano et al., 2002, Toscano et al., 2003). Interestingly, under similar experimental conditions no alteration at the phosphorylation state of CAMKII has been recorded (Toscano and Guilarte, 2005).

In primary hippocampal neurons exposed to 1 μM Pb^2+^ for 5 days during the period of synaptogenesis (DIV7–DIV12), both proBDNF protein levels and mBDNF were decreased with the latter to smaller extend (Neal et al., 2010). In the same in vitro model, Pb^2+^ also decreases dendritic proBDNF protein levels along the length of the dendrites and causes impairment of BDNF vesicle transport to sites of release in dendritic spines (Stansfield et al., 2012). Rat pups on PND 25 exposed to Pb^2+^ (180 and 375-ppm lead acetate in food for 30 days) demonstrated blood Pb^2+^ levels 5.8 to 10.3 μg/dl on PND 55 and show no change at gene levels of BDNF (Schneider et al., 2012). In mouse embryonic stem cells (ESCs), *Bdnf* exon IV has been found to be down-regulated in cells treated with 0.1 μM Pb^2+^, whereas *Bdnf* exon IX has been found up-regulated (Sánchez-Martín et al., 2013).

### 3.4 KER: reduced release of BDNF (KE upstream) leads to aberrant dendritic morphology (KE downstream)

#### 3.4.1 KE downstream: Aberrant dendritic morphology

After becoming post-mitotic and during the differentiation process, neuronal cells undergo lengthening, branching, dendrite and dendritic spine formation (Scott and Luo, 2001). In human, dendrites appear as early as 13.5 weeks gestation in neurons while arborization begins only after 26 weeks (Mrzljak et al., 1988, Mrzljak et al., 1990). In rodents, during the first postnatal week, both pyramidal and nonpyramidal neurons go through extensive and fast dendrite growth, branching, and elaboration. Dendrite arbor's capacity and complexity continue to increase in the second and third postnatal week, however, much slower. During the same developmental window, dendritic spines begin to appear as a group that then mature (Dailey and Smith, 1996). At this final stage of dendrite growth, a neuron possesses a dynamic dendrite tree, which has a greater potential for connectivity and synapse creation because of dendritic spine formation and maturation. Postsynaptic density-95 (PSD-95), a protein involved in dendritic spine maturation and clustering of synaptic signalling proteins, plays a critical role in regulating dendrite outgrowth and branching, independent of its synaptic functions (Kornau et al., 1995).

Functionally, dendrites serve as post-synaptic part of a synapse, playing a critical role in the processing of information transmitted through synapses. They receive the majority of synaptic inputs comparing to the soma or the axon. Postsynaptic activity is closely related to the properties of the dendritic arbor itself, implying that the dendrites strongly influence and control synaptic transmission and plasticity (Sjöström et al., 2008; Segal, 2017).

#### 3.4.2 WoE evaluation

##### 3.4.2.1 Biological plausibility for this KER

There is extensive scientific understanding of the functional mechanistic relationship between KEup Reduced release of BDNF and the following KEdown Aberrant Dendritic Morphology (Fig. 3D). After activation of tyrosine kinase B (TrkB) receptors by BDNF, proteins such as Arc, Homer2, LIMK1 are released (Schratt et al., 2004; Yin et al., 2002). These proteins that are known to promote actin polymerization lead to enlargement of dendritic spine heads (Sala et al., 2001). It has also been shown that BDNF promotes dendritic spine formation by interacting with Wnt signalling. Indeed, Wnt signalling inhibition in cultured cortical neurons causes disruption in dendritic spine development, reduction in dendritic arbor size, complexity and blockage of BDNF-induced dendritic spine formation and maturation (Hiester et al., 2013). In addition, it has been shown that the inhibition of BDNF synthesis reduces the size of spine heads and impairs LTP (An et al., 2008; Waterhouse and Xu, 2009). BDNF has been characterised as a critical factor in promoting dendritic morphogenesis in various types of neurons (Jan and Jan, 2010; Park and Poo, 2013). BDNF synthesised in dendrites is known to regulate the morphology of spines (Tyler and Pozzo-Miller, 2003; An et al., 2008). For example, spines in the absence of spontaneous electrical activity are significantly smaller than normal (Harvey et al., 2008). On the other hand, simultaneous electrical activity and Glu release increase the size of the spine head, which has been shown to be dependent on BDNF presence (Tanaka et al., 2008).

Mice caring the Val66Met mutation of Bdnf gene show dendritic arborization defects in the hippocampus. Interestingly, human subjects with the Val66Met SNP demonstrate similar anatomical features (reviewed in Cohen and Greenberg, 2008).

##### 3.4.2.2 Empirical support for linkage

There is no direct evidence linking decreased BDNF levels to aberrant dendritic morphology as they have not been ever measured both in the same study after exposure to stressors. However, several studies provide empirical support for this KER. The reduction in the length of dendritic processes and the number of dendritic branches in hippocampal dentate granule cells was demonstrated after developmental Pb^2+^ exposure of Long-Evans hooded rat pups (Alfano and Petit, 1982). More recently, it has been shown that the chronic exposure of rats to environmentally relevant levels (Pb^2+^ blood levels 25.8 ± 1.28 μg/dl) during early life alters cell morphology in the dentate gyrus as immature granule cells immunelabelled with doublecortin display aberrant dendritic morphology (Verina et al., 2007).

Exposure of rats to Pb^2+^ that was initiated at embryonic phase and terminated at PND 21 revealed that at PND 14 (Pb^2+^ concentration in the hippocampus 0.249 ± 0.06 μg/g) and PND 21 (Pb^2+^ concentration in the hippocampus 0.471 ± 0.11 μg/g) the number of dendritic spine on hippocampal CA1 area decreases by 32.83% and 24.11%, respectively (Hu et al., 2014). In separate in vivo study, low blood levels of Pb^2+^ (10 ± 1.28 μg/dl) in similar age of rats has led to significant decrease of BDNF concentration (pg/mg protein) by 39% in forebrain cortex and by 29% in hippocampus (Baranowska-Bosiacka et al., 2013).

In cultured rat hippocampal neurons, low levels of Pb^2+^ (0.1 and 1 μM) cause reduction of dendritic spine density in a concentration-dependent manner (Hu et al., 2014). In the same in vitro model, exposure to 1 μM Pb^2+^ for 5 days during the period of synaptogenesis (DIV7–DIV12), significantly reduces proBDNF protein and extracellular levels of mBDNF (Neal et al., 2010). When mouse embryonic stem cells are differentiated into neurons, exposure to lead (II) acetate causes reduction in the percentage of microtubule-associated protein 2 (MAP-2)-positive cells and in the mRNA levels of MAP-2, which is a dendrite marker, in a concentration-dependent manner (Baek et al., 2011).

### 3.5 KER: reduced release of BDNF (KE upstream) leads to reduced presynaptic release of glutamate (KE downstream)

#### 3.5.1 KE downstream: reduced presynaptic release of glutamate

Glu is an amino acid, the main excitatory neurotransmitter that is stored in presynaptic vesicles by the action of vesicular glutamate transporters (VGLUTs). Glu is mainly released from the presynaptic vesicles in a Ca^2+^-dependent mechanism that involves N- and P/Q-type voltage-dependent Ca^2+^ channels, closely linked to vesicle docking sites (reviewed in Meldrum, 2000). The pre-synaptic release of Glu is controlled by a wide range of presynaptic receptors that are not only glutamatergic like Group II and Group III of glutamate metabotropic receptors but also cholinergic such as nicotinic and muscarinic, adenosine (A1), kappa opioid, γ-aminobutyric acid (GABA)B, cholecystokinin and neuropeptide Y (Y2) receptors. Following its release, Glu exerts its effects via ionotropic and metabotropic receptors. Although Glu is available for binding to receptors for a short time, NMDA receptors show high affinity for this specific neurotransmitter that causes their activation compared to other receptors. Astrocytes play an important role in removing glutamate from synaptic cleft (Verkhratsky and Butt, 2007).

During development, glutamate is known to play important role as it regulates neurogenesis, neurite outgrowth, synaptogenesis and apoptosis (Meldrum, 2000; Mattson, 2008).

#### 3.5.2 WoE evaluation

##### 3.5.2.1 Biological plausibility *for this KER*

The functional mechanistic relationship between KEup *Reduced release of BDNF* and the following KEdown *Reduced presynaptic release of glutamae* is not completely established (Fig. 3E). It has been shown that presynaptically activated TrkB receptors by BDNF enhances Glu release and increases the frequency of miniature excitatory postsynaptic currents (mEPSCs) in hippocampal neurons of rat (Minichiello, 2009). It has been reported that BDNF rapidly induces Glu transporter-mediated glutamate release via phospholipase C-γ (PLC-γ)/Ca^2+^ signalling. In contrast, it has been shown that antidepressants enhance PLC-γ/Ca^2+^ signalling leading to reduced levels of BDNF that cause decreased Glu release (Numakawa et al., 2002; Yagasaki et al., 2006). However, in heterozygous BDNF-knockout (BDNF+/−) mice it has been demonstrated that the reduced BDNF levels did not affect presynaptic Glu release (Meis et al., 2012).

##### 3.5.2.2 Empirical support for linkage

In cortical cultured neurons obtained from rat pups PND 2-3, BDNF fails to induce Glu release at DIV 3 and 4. However, after 5 days in vitro culture or later (DIV 6-9), BDNF (5 or 100 ng/ml) induces significant Glu release within 1 min after exogenous BDNF application (Numakawa et al., 2002). No studies have been found in the literature measuring both KEs after exposure to the same stressors. Interestingly, proton magnetic resonance spectroscopy in adults with childhood lead exposure shows decrease in Glu and glutamine in vermis and in parietal white matter of the brain (Cecil et al., 2011).

### 3.6 KER: reduced release of BDNF (KE upstream) leads to neuronal cell death (KE downstream)

#### 3.6.1 KE downstream: cell injury/death

Cell death can be manifested either as apoptosis that involves shrinkage, nuclear disassembly, and fragmentation of the cell into discrete bodies with intact plasma membranes, or as necrosis that involves the loss of plasma membrane integrity (Nicotera et al., 1999). An important feature of apoptosis is the requirement for adenosine triphosphate (ATP) to initiate the execution phase. In contrast, necrotic cell death is characterised by cell swelling and lysis. This is usually a consequence of profound loss of mitochondrial function, resulting in ATP depletion, leading to loss of ion homeostasis and increased Ca^2+^ levels. The latter activates a number of nonspecific hydrolases (i.e., proteases, nucleases, and phospholipases) as well as calcium dependent kinases. Activation of calpain I, the Ca^2+^-dependent cysteine protease cleaves the death-promoting Bcl-2 family members Bid and Bax which translocate to mitochondrial membranes, resulting in release of truncated apoptosis-inducing factor (tAIF), cytochrome *c* and endonuclease in the case of Bid and cytocrome c in the case of Bax (Momeni, 2011). Two alternative pathways - either extrinsic (receptor-mediated) or intrinsic (mitochondria-mediated) - lead to apoptotic cell death. The initiation of apoptosis begins either at the plasma membrane with the binding of TNF or FasL to their cognate receptors or within the cell through mitochondria mediated pathways of apoptosis.

#### 3.6.2 WoE evaluation

##### 3.6.2.1 Biological plausibility for this KER

There is extensive scientific understanding of the functional mechanistic relationship between KEup Reduced release of BDNF and the following KEdown Neuronal cell death. BDNF is involved in the apoptosis occurring in developing neurons through two distinct mechanisms (Bernd, 2008) (Fig. 3F). mBDNF can trigger prosurvival signalling after binding to TrkB receptor through inactivation of components of the cell death machinery and also through activation of the transcription factor cAMP-response element binding protein (CREB), which drives expression of the pro-survival Bcl-2 gene (West et al., 2001). On the other hand, proBDNF binds to the p75 neurotrophin receptor (p75NTR) and activates RhoA that regulates actin cytoskeleton polymerization resulting in apoptosis (Miller and Kaplan, 2001; Murray and Holmes, 2011). It is proved that reduced levels of BDNF can severely interfere with the survival pathway of neurons in different brain regions, leading to cell death (Miller and Kaplan, 2001; Murray and Holmes, 2011).

BDNF mRNA levels dramatically increase between embryonic days 11 to 13 during rat brain development, playing important role in survival and neuronal differentiation (reviewed in Murray and Holmes, 2011). The latter has been supported by transgenic experiments where BNDF^−/−^ mice demonstrated a dramatic increase in cell death among developing granule cells leading to impaired development of the cerebellar cortex layers (Schwartz et al., 1997). The neuroprotective role of BDNF has been further supported by the observed correlation between elevated BDNF protein levels and resistance to ischemic damage in hippocampus in vivo (Kokaia et al., 1996) and K^+^ rich medium-induced apoptosis in vitro (Kubo et al., 1995).

Several studies addressing apoptosis mainly in the developing cerebral cortex have shown that other mechanism besides neurotrophic factors may be involved. Cytokines, as well as neurotransmitters can potentially activate a number of intracellular proteins that execute cell death (Henderson, 1996; Kroemer et al., 2009), meaning that further branches to this AOP might be added in the future.

##### 3.6.2.2 Empirical support for linkage

Several in vitro and in vivo studies on cortical neurons have demonstrated that the survival of developing neurons is closely related with the activation of the NMDA receptors and subsequent BDNF synthesis/release, fully supporting the BDNF role as a critical neurotrophic factor (Yoon et al., 2003; Hansen et al., 2004). However, there are no studies in scientific literature reporting on change in both KEs, measured in the same experiment, following exposure to Pb^2+^.

Neonatal mice exposed to Pb^2+^ (350 mg/kg lead twice every 4 h) and sacrificed after 8–24 h have shown increased apoptotic neurodegeneration in comparison to controls. This effect has been recorded only in animals treated with Pb^2+^ at PND 7, but not at PND 14 (Dribben et al., 2011), confirming the importance of the time of exposure during development in order for Pb^2+^ to induce apoptosis. Two to four weeks old rats treated for 7 days with 15 mg/kg daily dose of lead acetate show increased apoptosis in hippocampus (Sharifi et al., 2002). In older rats (30 PND), it has also been shown that Pb^2+^ (2, 20 and 200 mg/kg/d) can induce apoptosis (Liu et al., 2010). However, in contrast to the first two in vivo studies, the animals in these experiments were old enough to evaluate the most sensitive window of vulnerability of developing neurons to Pb^2+^ exposure (Liu et al., 2010), confirming that Pb^2+^ treatment during synaptogenesis leads to significant neuronal cell apoptosis. In vitro evidence of lead-induced apoptosis has also been studied in cultured rat cerebellar neurons (Oberto et al., 1996), and hippocampal neurons (Niu et al., 2002). However, a number of studies demonstrate that deletion of BDNF does not lead to significant apoptotic cell death of neurons in the developing CNS (reviewed in Dekkers et al., 2013). In an in vivo Pb^2+^ exposure study, where female rats received 1500 ppm prior, during breeding and lactation shows no changes at mRNA levels of BDNF in different hippocampus section derived from their pups (Guilarte et al., 2003). Regarding Pb^2+^, the pre- and neonatal exposure of rats to Pb^2+^ (Pb^2+^ blood levels below 10 μg/dl) show a decreased number of hippocampal neurons but no morphological or molecular features of severe apoptosis or necrosis have been detected in tested brains (Baranowska-Bosiacka et al., 2013), possibly due to effective microglial phagocytosis. After exposure to led reduced concentration (pg/mg protein) of BDNF in brain homogenates has been recorded in forebrain cortex (39%) and hippocampus (29%) (Baranowska-Bosiacka et al., 2013). In other studies, pregnant rats have been exposed to lead acetate (0.2% in the drinking water) after giving birth until PND 20 reaching blood Pb^2+^ levels in pups of 80 μg/dl. In these animals, hippocampus was the most sensitive to Pb^2+^ exposure, showing an increase of caspase-3 mRNA as early as PND12 (Chao et al., 2007).

### 3.7 KER: aberrant dendritic morphology (KE upstream) leads to decreased synaptogenesis (KE downstream)

#### 3.7.1 KE downstream: decreased synaptogenesis

Synaptogenesis follows axonal migration, during which presynaptic and postsynaptic differentiation occurs (Garner et al., 2002). “Synaptic assembly” refers to the mechanisms involved in recruitment of molecules required for differentiation, stabilization and maturation of synapse (Colón-Ramos, 2009). In human, synaptogenesis does not happen at the same time in all brain regions, as the prefrontal cortex lags behind in terms of synapse formation compared to the auditory and visual cortices. In contrast, synaptogenesis appears to proceed concurrently in different brain areas for rhesus monkey (Erecinska et al., 2004).

The period of rapid synaptogenesis or the so-called brain growth spurt is considered one of the most important processes for neuronal networking that take place during brain development (Garner et al., 2002). This process plays a vital role in synaptic plasticity, learning and memory and adaptation throughout life.

#### 3.7.2 WoE evaluation

##### 3.7.2.1 Biological plausibility for this KER

It is well-established that loss of dendritic spine density and aberrant dendrite branch complexity leads to loss of synapse formation (Fig. 3G). Indeed, huge amount of research has been performed on dendrite arbour, dendritic spines and the molecular components of these structures that led to the elucidation of their role in higher order brain functions, including learning and memory (Sjöström et al., 2008). The appearance of extensive dendritic arbor and new spines coincides with synapse formation (Zito et al., 2004). Zhang and Benson (2001) have investigated the role of actin (the main component of dendritic spines) during the early stages of neuronal development by introducing an actin depolymerization protein named latrunculin A and conducting fluorescent imaging of synapse formation. At the early stages of neuronal development, it has been reported that the depolymerisation of filamentous actin (F-actin) significantly reduces the number of stable synapses and the presence of postsynaptic proteins (PSD-95, neuroligins and Bassoon). Most importantly, pre- and postsynaptic vesicles needed for synaptogenesis have not been found at contact sites as a result of depolymerisation of F-actin (Zhang and Benson, 2001), proving the important role of dendritic arbor in synapse formation.

##### 3.7.2.2 Empirical support for linkage

Many studies have indicated that synaptogenesis and dendritic spine formation happen in any order (Bhatt et al., 2009; McAllister, 2007).Newborn rats exposed to 10 mg/ml of lead acetate from PND 2 up to PND 20 and 56 demonstrated significant decrease in spine density as shown in Golgi staining of hippocampal pyramidal neurons in CA1 region (Kiraly and Jones, 1982).

### 3.8 KER: reduced presynaptic release of glutamate (KE upstream) directly leads to decreased synaptogenesis (KE downstream)

KE upstream: Reduced presynaptic release of glutamate (described in Section 3.5.1)
KE downstream: Decreased synaptogenesis (described in Section 3.7.1)

#### 3.8.1 WoE evaluation

##### 3.8.1.1 Biological plausibility for this KER

Based on the exiting data biological plausibility for this KER was evaluated as strong and empirical support as moderate (Fig. 3H). It is well documented that the presynaptic release of Glu causes activation of NMDA receptors and initiates synaptogenesis through activation of downstream signalling pathways required for synapse formation (e.g. Ghiani et al., 2007). Lack or reduced presynaptic Glu release affects the transcription and translation of molecules required in synaptogenesis (Ghiani et al., 2007). The NMDA receptor activation by Glu during development increases calcium influx, which acts as a secondary signal for synaptogenesis. Eventually, immediate early genes (IEG) activation is triggered by transcription factors and the proteins required for synapse formation are produced (reviewed in Ghiani et al., 2007). Glu released from entorhinal cortex neurons has been shown to promote synaptogenesis in developing targeted hippocampal neurons (Mattson et al., 1988). Similarly, Glu has been found to regulate synaptogenesis in the developing visual system of frogs (Cline and Constantine-Paton, 1990).

The ratio of synaptic NR2B over NR2A NMDAR subunits controls dendritic spine motility and synaptogenesis. The intracellular C terminus of NR2 recruits the signalling and scaffolding molecules necessary for proper synaptogenesis (Gambrill and Barria, 2011).

##### 3.8.1.2 Empirical support for linkage

Reduced presynaptic release of Glu is linked to LTP, which is considered the functional measurement of synaptogenesis. Indeed, measures of presynaptic function at glutamatergic synapses in chronically exposed animals have produced results that can be related to the effects of Pb^2+^ on Glu and LTP. Animals exposed to 0.2% Pb^2+^ show decrease of hippocampal Glu release, diminishing the magnitude of hippocampal LTP (Gilbert et al., 1996). Microdialysis experiment in animals exhibiting blood Pb^2+^ levels of 30–40 μg/100 ml show diminished depolarization-induced hippocampal Glu release (Lasley et al., 1999).

In another study, experiments in rats continuously exposed to 0.1–0.5% Pb^2+^ in the drinking water beginning at gestational day 15–16 resulted in decreased hippocampal Glu release (Lasley and Gilbert, 2002), confirmed also in hippocampal cultures and brain slices exposed to Pb^2+^ (Braga et al., 1999; Xiao et al., 2006). The chronic in vivo exposure to Pb^2+^ during development results in a marked inhibition of Schaffer-collateral-CA1 synaptic transmission by inhibiting vesicular release of Glu, an effect that is not associated with a persistent change in presynaptic calcium entry (Zhang et al., 2015).

### 3.9 KER: Neuronal cell death (KE upstream) leads to decreased synaptogenesis (KE downstream)

KE upstream: Cell injury/death (described in Section 3.6.1)
KE downstream: Decreased synaptogenesis (described in Section 3.7.1)

#### 3.9.1 WoE evaluation

##### 3.9.1.1 Biological plausibility for this KER

Based on the current mechanistic knowledge biological plausibility and empirical support for this KER was evaluated as moderate (Fig. 3I). Under physiological conditions, in the developing nervous system, apoptosis occurs during the process of synaptogenesis, where competition leads to the loss of excess neurons and to the connection of the appropriate neurons (Buss et al., 2006; Mennerick and Zorumski, 2000). However, increased apoptosis leads to defective synaptogenesis as the reduced number of neurons (besides the ones that have been already eliminated through the physiological process of apoptosis) decreases dendritic fields for receiving synaptic inputs from incoming axons. At the same time the loss of neurons also means that there are less axons to establish synaptic contacts (Olney, 2014), leading to reduced synaptogenesis and decreased neuronal networking. Recently, Dekkers et al. (2013) have reviewed how the apoptotic machinery in developing brain regulate synapse formation and neuronal connectivity. For example, caspase activation is known to be required for axon pruning during brain development to generate neuronal network. In *Drosophila melanogaster* and in mammalian neurons components of apoptotic machinery are involved in axonal degeneration that can consequently interfere with synapse formation (reviewed in Dekkers et al., 2013).

##### 3.9.1.2 Empirical support for linkage

Synaptogenesis and refinement of the cortical network precedes the programmed cells death of neurons during development (Innocenti and Price, 2005). Elevated blood Pb^2+^ concentrations in new-born rats prenatally exposed to 30 or 200 mg/l Pb^2+^ causes postnatally delay in synaptogenesis (McCauley et al., 1982). In this study, Pb^2+^ treatment depresses synaptic number in pups at PND 11 to 15 but not in older pups (McCauley et al., 1982). In rat hippocampal primary cultures, Pb^2+^ treatment has no effect on PSD95 puncta density nor has any effect on Synapsin Ia/b total gray value, puncta density, and integrated intensity but reduces the phosphorylation of Synapsin Ia/b (Stansfield et al., 2012). Pb^2+^ exposure also represses the expression of presynaptic vesicular proteins implicated in neurotransmitter release, such as synaptobrevin (VAMP1) and synaptophysin (SYN) (Neal et al., 2010). In mouse embryonic stem cells (ESCs) cultured in 3D aggregates, the treatment with Pb^2+^ causes around 25% of cell loss (Sánchez-Martín et al., 2013). In in vivo model, Pb^2+^ causes downregulation of Syn1 gene expression in the hippocampus of male offspring (PND 60) derived from female mice exposed to lead acetate in drinking water from 8 weeks prior to mating, through gestation and until postnatal day PND 10 (Sánchez-Martín et al., 2013).

### 3.10 KER: decreased synaptogenesis (KE upstream leads to decreased neuronal network function (KE downstream)

KE upstream: Decreased synaptogenesis (described in Section 3.7.1)

#### 3.10.1 KE downstream: Decreased neuronal network function

The neuronal network in developing brain shows a slow maturation and a transient passage from spontaneous, long-duration action potentials to synaptically-triggered, short-duration action potentials. At this stage, the neuronal network is characterised by “hyperexcitability”, which is related to the increased number of local circuit recurrent excitatory synapses and the lack of γ-amino-butyric acid A (GABAA)-mediated inhibitory function that appears much later. This “hyperexcitability” disappears with maturation when pairing of the pre- and postsynaptic partners occurs and synapses are formed generating population of postsynaptic potentials and population of spikes followed by developmental GABA switch (reviewed in Erecinska et al., 2004). Glutamatergic neurotransmission is dominant at early stages of development and NMDA receptor-mediated synaptic currents are far more times longer than those in maturation, allowing more calcium to enter the neurons. The processes that are involved in increased calcium influx and the subsequent intracellular events seem to play a critical role in establishment of wiring of neuronal circuits and strengthening of synaptic connections during development (Erecinska et al., 2004). Neurons that do not receive glutaminergic stimulation are undergoing developmental apoptosis. The development of neuronal networks can be distinguished into two phases: an early “establishment” phase of neuronal connections, where activity-dependent and independent mechanisms could operate, and a later “maintenance” phase, which appears to be controlled by neuronal activity (Yuste et al., 1992). These neuronal networks facilitate information flow that is necessary to produce complex behaviours, including learning and memory (Mayford et al., 2012).

#### 3.10.2 WoE evaluation

##### 3.10.2.1 Biological plausibility for this KER

The current mechanistic knowledge of biological plausibility and empirical support for this KER was evaluated as moderate (Fig. 3J). The ability of a neuron to communicate is based on neuronal network formation that relies on functional synapse establishment (Colón-Ramos, 2009). The main roles of synapses are the regulation of intercellular communication in the nervous system, and the information flow within neuronal networks. The connectivity and functionality of neuronal networks depends on where and when synapses are formed. Therefore, the decreased synapse formation during the process of synaptogenesis is critical and leads to decrease of neuronal network formation and function in the adult brain. The developmental period of synaptogenesis is critical for the formation of the basic circuitry of the nervous system, although neurons are able to form new synapses throughout life (Rodier, 1995). The brain electrical activity dependence on synapse formation is critical for proper neuronal communication. Alterations in synaptic connectivity lead to refinement of neuronal networks during development (Cline and Haas, 2008). Indeed, knockdown of PSD-95 arrests the functional and morphological development of glutamatergic synapses (Ehrlich et al., 2007).

##### 3.10.2.2 Empirical support for linkage

At low Pb^2+^ levels (less than 30 μg/dl), slow cortical potentials have been observed to be positive in children under five years old but negative in children over five years. However, age-related polarity reversal has been observed in children with higher Pb^2+^ levels (Otto and Reiter, 1984). In experiments carried out in Wistar rats that have been fed with lead acetate (400 micrograms lead/g body weight/day) from PND 2 until PND 60, the electroencephalogram (EEG) findings show statistically significant reduction in the delta, theta, alpha and beta band of EEG spectral power in motor cortex and hippocampus with the exception of the delta and beta bands power of motor cortex in wakeful state (Kumar and Desiraju, 1992).

Male Sprague-Dawley rats have been exposed to Pb^2+^ from parturition to weaning though their dams' milk that received drinking water containing 1.0, 2.5, or 5.0 mg/ml lead acetate (McCarren and Eccles, 1983). Beginning from 15 weeks of age, the characteristics of the electrically elicited hippocampal after discharge (AD) and its alteration by phenytoin (PHT) showed significant increase in primary AD duration only in the animals exposed to the higher dose of Pb^2+^, whereas all groups responded to PHT with increases in primary AD duration (McCarren and Eccles, 1983).

In primary rat cortical neurons (12–22 DIV), Pb^2+^ (50 μM) slightly increases mean firing rate (MFR) as measured by micro-electrode-array (MEA) technology (McConnell et al., 2012).

### 3.11 KER: decreased neuronal network function (KE upstream) leads to impairment of learning and memory (KE downstream)

KE upstream: Decreased neuronal network function (described in Section 3.10.1)
KE downstream: impairment of learning and memory, the adverse outcome (AO)

#### 3.11.1 AO, learning and memory

Learning can be defined as the process by which new information is acquired to establish knowledge by systematic study or by trial and error (Ono, 2009). Two types of learning are considered in neurobehavioral studies: a) associative learning and b) non-associative learning. The memory to be formed requires acquisition, retention and retrieval of information in the brain, which is characterised by the non-conscious recall of information (Ono, 2009). Memory is considered very important as it allows the subjects to access the past, to form experience and consequently to acquire skills for surviving purposes. There are three main categories of memory, including sensory memory, short-term or working memory (up to a few hours) and long-term memory (up to several days or even much longer). At the cellular level the storage of long-term memory is associated with increased gene expression and protein synthesis as well as formation of novel synaptic connections (Lynch, 2004).

Learning-related processes require neuronal networks to detect correlations between events in the environment and store these as changes in synaptic strength (Abbott and Nelson, 2000). Long-term potentiation (LTP) and long-term depression (LTD) are two fundamental processes involved in cognitive functions (Abbott and Nelson, 2000; Malenka and Bear, 2004), which respectively, strengthen synaptic inputs that are effective at depolarizing the postsynaptic neuron and weaken inputs that are not, thus reinforcing useful pathways in the brain. The best characterisation form of LTP occurs in the CA1 region of the hippocampus, in which LTP is initiated by transient activation of receptors and is expressed as a persistent increase in synaptic transmission through AMPA receptors followed by activation of NMDARs. This increase is due, at least in part, to a postsynaptic modification of AMPA-receptor function; this modification could be caused by an increase in the number of receptors, their open probability, their kinetics or their single-channel conductance. Summing up activity-dependent alteration in synaptic strength is a fundamental property of the vertebrate central nervous system that underlies learning and memory processes.

It is appropriate to state that while much emphasis has been given on the key role of the hippocampus in memory, it would probably be simplistic to attribute memory deficits solely to hippocampal damage (Barker and Warburton, 2011). There is substantial evidence that fundamental memory functions are not mediated by hippocampus alone but require a network that includes, in addition to the hippocampus, anterior thalamic nuclei, mammillary bodies cortex, cerebellum and basal ganglia (Doya, 2000; Mitchell et al., 2002; Toscano and Guilarte, 2005). Each of these brain structures can be potentially damaged leading to more or less severe impairment of learning and memory.

#### 3.11.2 WoE evaluation

##### 3.11.2.1 Biological plausibility for this KER

Based on the current mechanistic understanding and evaluable empirical data biological plausibility and empirical support for this KER was evaluated as moderate (Fig. 3K). Learning and memory is one of the outcomes of the functional expression of neurons and neuronal networks. Damage or destruction of neurons by a chemical during synaptogenesis when the process of synapses formation takes place, integration and formation of neuronal networks could be impaired causing derange of the synaptic organisation and function. Such changes in the neuronal network formation could lead to subsequent impairment of learning and memory processes. Exposure to the potential developmental toxicants during neuronal differentiation and synaptogenesis will increase risk of functional neuronal network damage leading to learning and memory impairment.

Long-term potentiation (LTP) is a long-lasting increase in synaptic efficacy, and its discovery suggested that changes in synaptic strength could provide the substrate for learning and memory (reviewed in Lynch, 2004; Sweatt, 2016). Moreover, LTP is intimately related to the theta rhythm, an oscillation long associated with learning. Learning-induced enhancement in neuronal excitability, a measurement of neuronal network function, has also been shown in hippocampal neurons following classical conditioning in several experimental approaches (reviewed in Saar and Barkai, 2003). On the other hand, memory requires the increase in magnitude of an excitatory postsynaptic currents (EPSCs) to be developed quickly and to be persistent for few weeks at least without disturbing already potentiated contacts. Once again, a substantial body of evidence have demonstrated that tight connection between LTP and diverse instances of memory exist (reviewed in Lynch, 2004).

##### 3.11.2.2 Empirical support for linkage

A series of important findings suggest that the biochemical changes that happen after induction of LTP also occur during memory acquisition, showing temporality between the two KEs (reviewed in Lynch, 2004). Furthermore, a review on Morris water maze (MWM) as a tool to investigate spatial learning and memory in laboratory rats also pointed out that the disconnection between neuronal networks rather than the brain damage of certain regions is responsible for the impairment of MWM performance (D'Hooge and De, 2001). Functional integrated neuronal networks that involve the coordination action of different brain regions are consequently important for spatial learning and MWM performance.

Exposure to low levels of Pb^2+^, during early development, has been implicated in long-lasting behavioral abnormalities and cognitive deficits in children (reviewed Neal and Guilarte, 2010) and experimental animals (Murphy and Regan, 1999; Moreira et al., 2001). Multiple lines of evidence suggest that Pb^2+^ can impair hippocampus-mediated learning in animal models (Toscano and Guilarte, 2005; Cao et al., 2008). Rat pups have been exposed to Pb^2+^ via their dams' drinking water from PND 1 to PND 21 and directly via drinking water from weaning until PND 30 (Jaako-Movits et al., 2005). At PND 60 and 80, the neurobehavioral assessment has revealed that developmental Pb^2+^ exposure induces persistent increase in the level of anxiety and inhibition of contextual fear conditioning (Jaako-Movits et al., 2005; Salinas and Huff, 2002), being in agreement with observations on humans. Indeed, children exposed to low levels of Pb^2+^ display attention deficit, increased emotional reactivity and impaired memory and learning (Finkelstein et al., 1998).

In experiments carried out in Wistar rats, fed with lead acetate from PND 2 until PND 60, EEG findings show statistically significant reduction in the delta, theta, alpha and beta band EEG spectral power in motor cortex and hippocampus (Kumar and Desiraju, 1992). After 40 days of recovery, animals have been assessed for their neurobehaviour and revealed that Pb^2+^ treated animals show more time and sessions in attaining criterion of learning than controls (Kumar and Desiraju, 1992). Further data obtained using animal behavioral techniques demonstrate that NMDA mediated synaptic transmission is decreased by Pb^2+^ exposure (Cory-Slechta, 1995; Cohen and Cory-Slechta, 1993, Cohen and Cory-Slechta, 1994). Selective impairment of learning and memory was also observed after blockade of long-term potentiation by AP5 (NMDAR antagonist) (Morris et al., 1986).

## 4 Overall assessment of the AOP for impairment of learning and memory abilities

The aim of the present AOP was to construct a pathway that captures the KEs and KERs that occur after binding of an antagonist to NMDA receptor in neurons during brain development (synaptogenesis) referring mainly to hippocampus and cortex, two fundamental brain structures involved in learning and memory formation. Recent study (Wang et al., 2014) reported that functional connectivity exists in cortical-hippocampal network and that the associative memory improves due to their cooperative function (Wang et al., 2014). Based on the supporting data for all KEs and KERs that are summarised in Table 1 and the modified Bradford-Hill considerations (including biological plausibility, essentiality of KEs, concordance of empirical data) (Users' Handbook (OECD, 2016a) confidence in the supporting data is considered as high.

The Biological plausibility for majority of the identified KERs is well documented as there is extensive mechanistic understanding supporting linkage between relevant KEs upstream and the KEs downstream, except for the KER between decreased neuronal network function that leads to learning and memory impairment. It is still unclear what modifications of neuronal circuits need to happen in order to trigger cognitive deficits, measurable in a learning and memory test (Mayford et al., 2012). This KER is only partially understood and further research is required to better explain the relationship between these two KEs.

Essentiality is also rated high because there is direct experimental evidence for most of the KEs showing that blocking KEs upstream prevents or attenuates the relevant KEs downstream and/or the AO (Table 1). Studies on transgenic animals and specifically designed inhibitors have provided direct evidence indicating the essentiality of the KEs in the mechanism that underpin LTP and underlie learning and memory processes in developing organisms. Memory enhancement studies also supported the essentiality of certain KEs by providing indirect evidence like for example in the case of the KE-Decreased neuronal network function for which is not experimentally possible to get direct evidence.

However, the empirical support for the majority of identified KERs cannot be rated high as in most occasions the KEup and KEdown of a KER have not been investigated simultaneously under the same experimental protocol. Furthermore, quantitative dose-response data on KERs are not available, therefore this AOP is mainly qualitative (not quantitative). Definition of thresholds for KEs upstream to be able to trigger KEs downstream is missing. For this reason WoE only for the first KER (Binding of antagonist toNMDA receptors leads to Inhibition of NMDARs) was rated as strong, for the second KER (Inhibition, NMDARs leads to Decreased, Calcium influx) as “moderate”, whereas all others.

**Table 1:**
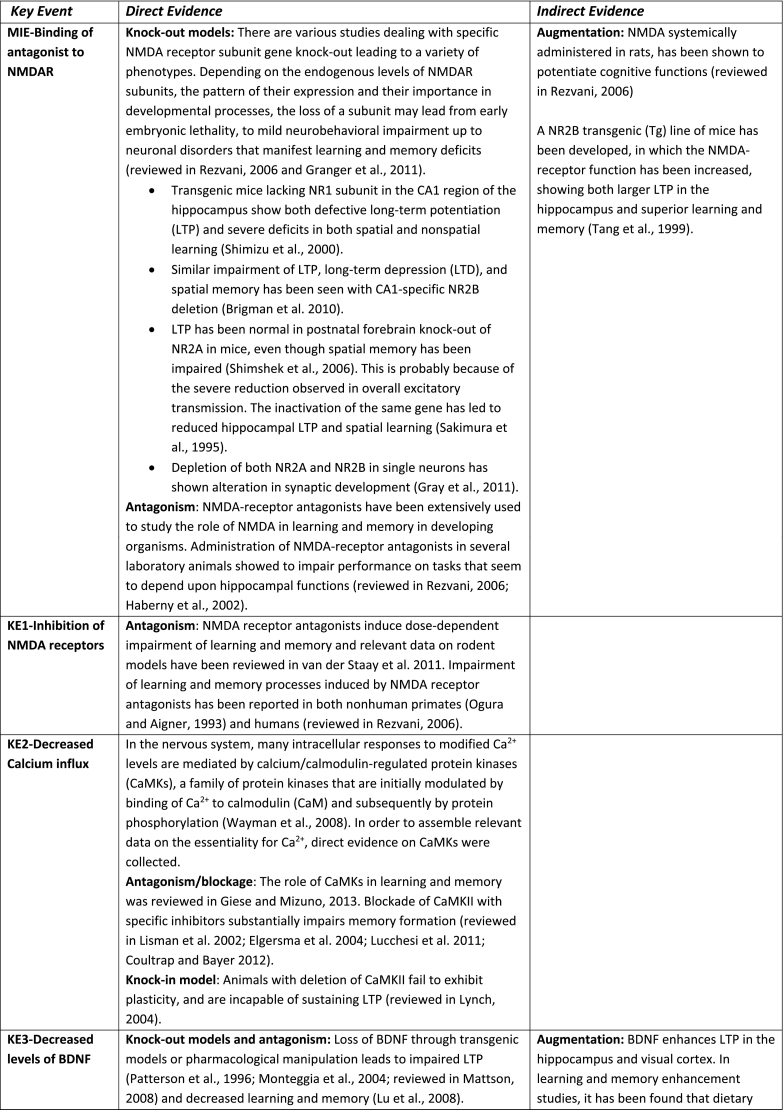

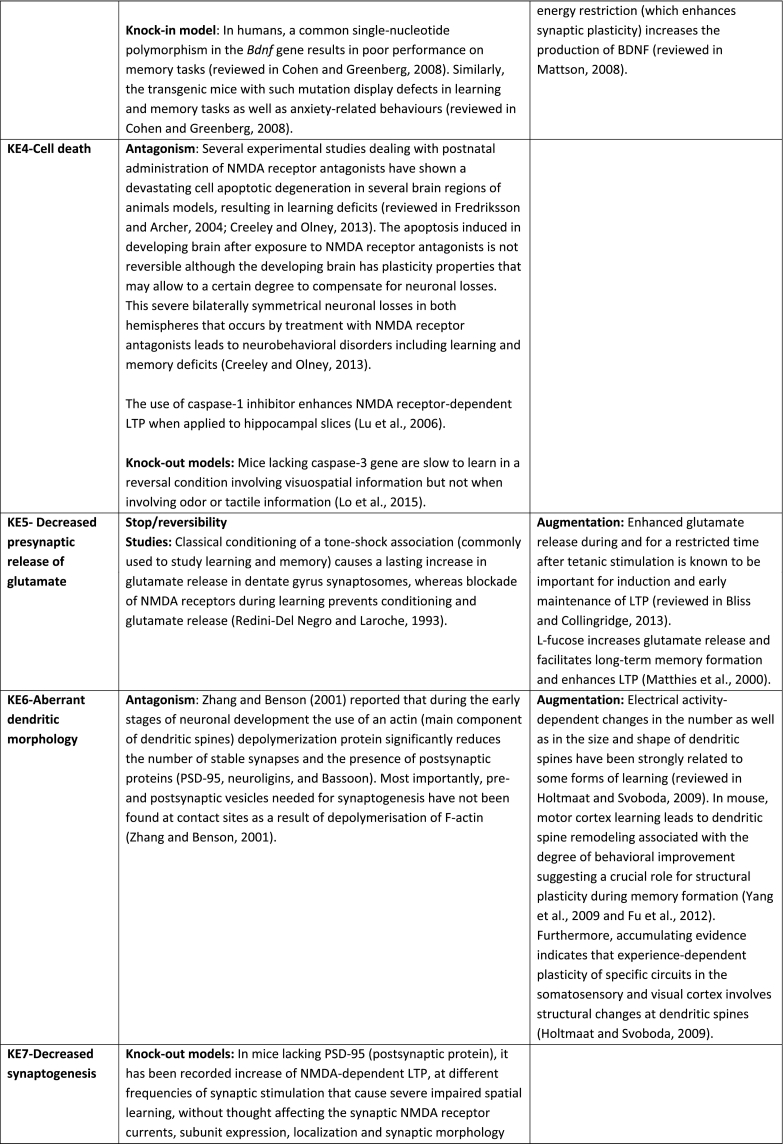

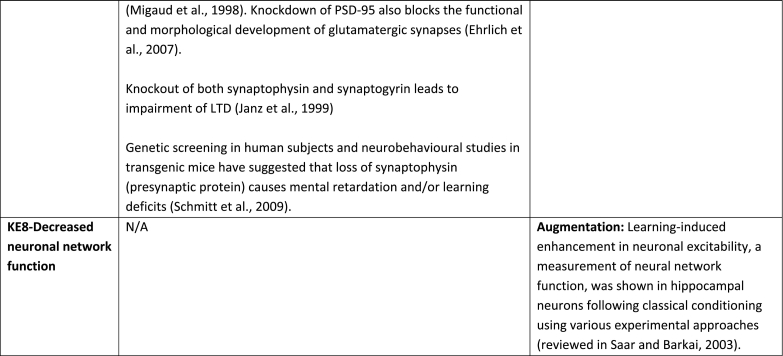
Assembly of the experimental data to support essentiality evaluation of Key Events (KEs) based on direct or indirect evidence. No contradictory results were found for any of the described KEs. Based on the supporting data, the weight of evidence for all KEs in the context of this AOP, overall, is considered as high.

Granger et al., 2011; Tang et al., 1999; Shimizu et al., 2000; Brigman et al., 2010; Shimshek et al., 2006; Sakimura et al., 1995; Gray et al., 2011; Haberny et al., 2002; Van der Staay et al., 2011; Ogura and Aigner, 1993; Wayman et al., 2008; Giese and Mizuno, 2013; Lisman et al., 2002; Elgersma et al., 2004; Lucchesi et al., 2011; Coultrap and Bayer, 2012; Patterson et al., 1996; Monteggia et al., 2004;Lu et al., 2008; Fredriksson and Archer, 2004; Creeley and Olney, 2013; Lu et al., 2006; Lo et al., 2015; Bliss and Collingridge, 2013; Redini-Del Negro and Laroche, 1993; Matthies et al., 2000; Holtmaat and Svoboda, 2009; Yang et al., 2009; Fu et al., 2012; Migaud et al., 1998; Janz et al., 1999; Schmitt et al., 2009.

## 5 Discussion

It is well understood and documented that learning and memory processes rely on the physiology of the NMDA receptor. If the function of this receptor is blocked during brain development (especially synaptogenesis) it can result in learning and memory impairment in children through a cascade of KEs described in this AOP.

Synaptogenesis is a fundamental process of neuronal network formation and if disturbed can results in neurodevelopmental disorders (Habela et al., 2016). Therefore, early life exposure to environmental pollutants is critical in determining whether a child's brain development will provide a strong or weak foundation for future learning abilities. Many factors impact children brain development such as poor nutrition, foetal exposure to infectious agents but it is well proved that exposure to toxic environmental chemicals such as lead can directly impair brain and neurological development in children. Published experimental data, including epidemiological studies (reviewed in Neal and Guilarte, 2010) strongly suggest that environmental chemicals contribute in children to the lowered IQ, learning disabilities, attention deficit hyperactivity disorder (ADHD) and, in particular, autism (McDonald and Paul, 2010; Grandjean and Landrigan, 2006, Grandjean and Landrigan, 2014). Lowered intelligence from early childhood exposure to lead exposure alone was estimated to result in about $675 million per year in income lost to those affected in Washington State (Report of Washington State Departments of Ecology and Health, 2009). Clearly learning and memory deficit contributes to learning disability of children who have difficulty in reading, writing and learning new things, significantly interfering with school achievement. The burden of these conditions for families and society includes financial costs related to special education, medical treatment, law enforcement, and the social and emotional toll on the children and caregivers. Therefore, much effort is undertaken, including AOP concept, to scientifically prove which environmental chemicals (singles and in mixture) could trigger a cascade of events leading to cognitive deficit in children.

Learning and memory is an important endpoint or regulatory relevance and a wide variety of tests to assess chemical effects on cognitive functions is available and used for the study of neurotoxicity in adult and young laboratory animals. Currently, neurotoxicity testing guidelines (OECD TG 424, 443, 426 and US EPA OCSPP 870.6300) require testing of learning and memory when DNT or neurotoxicity studies are triggered in order to comply with relevant US and EU regulations (Makris et al., 2009). However, for learning and memory assessment the guidelines methodology is flexible and its sensitivity varies, which may lead to some difficulties in test interpretation (Raffaele et al., 2010; EFSA PPR Panel Scientific Opinion, 2013; Gilbert et al., 2012). Additionally, the OECD DNT TG 426 and US EPA OCSPP 870.6300 are rarely performed since it is costly, time consuming, require high number of laboratory animals and might provide scientifically unreliable information (Crofton et al., 2011, Crofton et al., 2012; Bal-Price et al., 2012, Bal-Price et al., 2015a; Smirnova et al., 2014). Furthermore, these in vivo tests rely mainly on the read-outs of the final adverse effects by observing clinical signs, neurobehavioral performance and neuro-pathological changes recorded after animal exposure to chemicals without providing any mechanistic information on the underlying biological processes leading to the AO. This kind of information is provided within the AOP concept as illustrated in the described AOP. It is one of the first AOPs developed according to the OECD guidelines and underwent reviewing process by the scientists in the field and finally endorsed by the OECD Working Group of National Coordinators of the Test Guidelines Programme (WNT) and Working Party on r Hazard Assessment (WPHA).

AOPs can be used for different regulatory purposes, aiming to use mechanistic toxicological information in order to develop novel testing strategies such as integrated approaches to testing and assessment (IATA) (Tollefsen et al., 2014; OECD, 2016b; Sachana and Leinala, 2017). Indeed, this AOP can provide rational for in vitro assays selection (or development) that should be anchored to the KEs defined in this AOP since causative links described between the identified KEs would increase scientific confidence in such battery of tests (AOP-informed IATA) (Bal-Price and Meek, 2017). These assays should be based on mixed population of human neuronal and glial cells derived from induced pluripotent stem cells (avoiding extrapolation), permitting quantitative evaluation of the KEs (Fig. 1), particularly those close to AO including reduced levels of BDNF (measured by protein and mRNA expression), neuronal differentiation (e.g., measured by neurite outgrowth), synaptogenesis (measured by co-localization or synaptophysin (pre-) and PSD 95 as post-synaptic protein) and neuronal network function by the measurements of neuronal electrical activity. These in vitro assays are already well standardized and ready to be used. Some of the KEs presented in this AOP have already been identified as important endpoints for mapping of available in vitro DNT assays by EFSA (Fritsche et al., 2015; Fritsche, 2016).

Quantitative measurements of the identified KERs are urgently needed to determine the thresholds for upstream KEs to be able to trigger downstream KEs, moving this current qualitative AOP towards more quantitative AOPs. Such quantitative data, combined with a computational modeling to predict human toxicity, could potentially permit risk assessment of compounds working primary via this AOP.

IATA should be based on various sources of information, including not only in vitro methods but also other non-testing methods such as quantitative structure activity relationship (QSAR), read across and in silico modeling. Indeed, computational chemistry methods were already applied to develop QSAR model, which allows to predict the activity of potent competitive NMDA antagonists (MIE). First, various molecular parameters were calculated for a series of competitive NMDA antagonists with known activity to link the computationally calculated parameters to experimentally determined molecules activity. The developed QSAR model allows to predict the activity of a potent competitive NMDA antagonists before its synthesis since only theoretically determined molecular parameters are used for the prediction (Korkut and Varnali, 2003). Another approach was applied to develop a QSAR model for non- competitive antagonists of NMDA receptor has also been developed by studying a series of 48 substituted MK-801 derivatives, permitting to predict the inhibitory activity of a set of new designed compounds (Chtitaa et al., 2015). 2D- and 3D-QSAR models have also been developed to establish the structural requirements for pyrazine and related derivatives selective for NR2B subunit of NMDA receptor antagonists (Zambre et al., 2015). Moreover, AOP based QSAR models can also facilitate grouping of chemicals according to their biological activities (e.g., chemicals that trigger MIE or a particular KE) and subsequent development of read-across approach.

These QSAR models together with in vitro assays anchored to KEs of the described AOP should be included in IATA (AOP-informed IATA) that could serve as a tool for an initial chemical screening and prioritization to identify those with potential to cause learning and memory impairment in children.

However, this AOP represents one of many possible cascade of events leading to learning and memory impairment in children. Further development of AOPs, interconnected into network is required to have more comprehensive understanding of different toxicity pathways involved. Such AOPs network will facilitate identification of common KEs for multiple AOPs that should be considered as anchors for in vitro assays development, increasing probability of identifying potential DNT compounds, even if they cause toxicity through different pathways and triggered by various MIEs.

The present AOP can encourage the development of new in vitro test battery and the use of these alternatives to assess NMDAR inhibitors as chemicals with potential to induce impairment of children cognitive function and at the same time reduce the use of in vivo studies. In addition, the majority of KEs in this AOP has strong essentiality to induce the AO (impairment of learning and memory) and established adjacent relationships between them that would allow not only the development of testing methods that address these specific KEs but also the understanding of the relationship between the measured KEs and the AO. In addition, this AOP is expecting to make significant contribution to a recent international effort that aims to develop an OECD guidance document on DNT evaluation and accelerate the development and use of in vitro assays and other alternative tools capable of cost and time efficient testing of chemicals for their potential to disrupt the development of the nervous system.
